# Investigation of Specific Proteins Related to Different Types of Coronary Atherosclerosis

**DOI:** 10.3389/fcvm.2021.758035

**Published:** 2021-10-22

**Authors:** Heyu Meng, Jianjun Ruan, Yanqiu Chen, Zhaohan Yan, Kaiyao Shi, Xiangdong Li, Ping Yang, Fanbo Meng

**Affiliations:** Jilin Provincial Precision Medicine Key Laboratory for Cardiovascular Genetic Diagnosis (Jilin Provincial Engineering Laboratory for Endothelial Function and Genetic Diagnosis of Cardiovascular Disease, Jilin Provincial Molecular Biology Research Center for Precision Medicine of Major Cardiovascular Disease, Jilin Provincial Cardiovascular Research Institute), Department of Cardiology, China-Japan Union Hospital of Jilin University, Changchun, China

**Keywords:** atherosclerosis, albumin, SHBG sex hormone-binding globulin, APOC2, APOC3, APOC4, SAA4

## Abstract

**Objective:** Coronary heart disease (CHD) is a complex disease caused by multifaceted interaction between genetic and environmental factors, which makes identification of the most likely disease candidate proteins and their associated risk markers a big challenge. Atherosclerosis is presented by a broad spectrum of heart diseases, including stable coronary artery disease (SCAD) and acute myocardial infarction (AMI), which is the progressive stage of SCAD. As such, the correct and prompt diagnosis of atherosclerosis turns into imperative for precise and prompt disease diagnosis, treatment and prognosis.

**Methods:** The current work aims to look for specific protein markers for differential diagnosis of coronary atherosclerosis. Thirty male patients between 45 and 55 years diagnosed with atherosclerosis were analyzed by tandem mass tag (TMT) mass spectrometry. The study excluded those who were additionally diagnosed with hypertension and type 1 and 2 diabetes. The Mufuzz analysis was applied to select target proteins for precise and prompt diagnosis of atherosclerosis, most of which were most related to high lipid metabolism. The parallel reaction monitoring (PRM) was used to verify the selected target proteins. Finally, The receiver operating characteristic curve (ROC) was calculated by a random forest experiment.

**Results:** One thousand one hundred and forty seven proteins were identified in the TMT mass spectrometry, 907 of which were quantifiable. In the PRM study, six proteins related to lipid metabolism pathway were selected for verification and they were ALB, SHBG, APOC2, APOC3, APOC4, SAA4.

**Conclusion:** Through the detected specific changes in these six proteins, our results provide accuracy in atherosclerosis patients' diagnosis, especially in cases with varying types of the disease.

## Introduction

The coronary heart disease (CHD) is presented by a broad spectrum of heart diseases, including stable coronary artery disease (SCAD) and acute myocardial infarction (AMI), which is the progressive stage of SCAD (1). The pathogenesis of CHD includes epicardial atherosclerosis and plaque formation, arterial and microvascular spasms (2). The outcome is myocardial infarction, which is a complex process that begins with atherosclerosis, progresses to endothelial cell dysfunction in the coronary arteries, and eventually leads to narrowing of the blood vessels. The last impedes blood flow to the heart, thus leading to AMI (3–5). In both SCAD and AMI, the coronary atherosclerosis is the cause of the disease.

Atherosclerosis by itself is a chronic inflammatory disease of the arteries that leads to the calcification of the lesions in the intima layer. The calcification process can be exacerbated by plaque formation, which makes atherosclerosis a marker of CHD progression (6). Atherosclerosis is progressive with three types of specific changes that appear successively: lipid spots and stripes, atherosclerotic and thin-cap fibroatheroma, followed by complex alterations in the arteries (7, 8). The American College of Cardiology classifies these lesions into 6 categories (types) based on their progression (9, 10). Type I and II are the initial stages characterized by lipid spots, small yellow dots in the intima of the arteries with foam cell aggregation for a small range of macrophages. In the intima, there are smooth muscle cells and lipid droplets that are infiltrated by T cells. Type III is called the plaque prophase in which more extracellular lipid droplets are present and lipid nuclei are made between the smooth muscle cells layers of the inner and middle membrane, but the lipid pool is not yet formed. Type IV is the stage in which the atheromatous plaque is formed. Lipids are more concentrated, which is an indication that the lipid pool is shaped. The intima structure is destroyed, and the arteries' walls are deformed. Type V is characterized by the formation of thin-cap fibroatheroma. It is the most characteristic lesion of atherosclerosis with white plaques protruding into the arterial lumen and causing stenosis of the lumen. The intima of the plaque surface is destroyed and the lipid pool is enclosed by proliferating fibrous cap. Type VI is referred to as a complex type of atherosclerosis lesions, namely, severe lesions. It is characterized by fibrous plaque bleeding, necrosis, ulceration, calcification and mural thrombosis formation. Calcification is an accurate predictor of future cardiovascular events and a key factor in atherosclerosis (11). Different levels of calcium deposition are associated with the progression and severity of cardiovascular diseases (12). In the early stages of the disease, conventional diagnostic imaging methods cannot detect microscopic changes. As a result, most patients are diagnosed in the late stages when already huge calcifications are present. Currently, there is no treatment for the prevention or treatment of calcification in cardiovascular diseases, indicating the need for extensive study in this area (13, 14).

The occurrence of atherosclerotic heart disease is the result of complex interactions among environmental and genetic factors. Recent data show that smoking and stress can easily lead to cardiovascular diseases (15, 16). Though the genetic factors are uncontrollable, the change in certain environmental influences such as lifestyle and smoking habits holds the potential to improve cardiovascular symptoms (17, 18). Noteworthy, genetic factors account for 50% of the probability for development of atherosclerosis. Hence, the early acquisition of accurate genetic biomarkers for atherosclerosis makes patients' early diagnosis prompt and timely, thus leading to correct therapeutic decisions. Therefore, it is of great significance to search for novel molecular markers for early diagnosis, timely warning, early intervention and improvement of CHD prognosis (19, 20).

Here, through a proteomic approach, we aim to screen for unique differential proteins closely related to different types of coronary atherosclerosis. Our results show that multiple protein markers define the different stages of atherosclerosis. Six of them, namely ALB, SHBG, APOC2, APOC3, APOC4, and SAA4 have been further verified. The results show that these genes are involved in the lipid metabolism pathway and can be used in the diagnosis and discrimination among different types and severity of atherosclerosis.

## Materials and Methods

### Patients' Classification and Peripheral Blood Plasma Collection

#### Patients' Classification

This study is a retrospective study. All selected patients signed informed consent and met the Helsinki Declaration. All data identifying patient information have been deleted. Peripheral blood plasma samples of 30 male patients aged 45–55 who were hospitalized in the Department of Cardiovascular Medicine, China-Japan Union Hospital, Jilin University from June 2017 to August 2018 were used in the study. Patients with accompanying diagnoses such as hypertension and diabetes were excluded. All others were subdivided into three groups. Ten patients were classified in the AMI group (T3), 10 patients were enrolled in the SCAD (stable coronary artery disease) group (T2) and 10 cases fell into the control group (C1). Plasma collection in AMI group was first diagnosed and collected within 12 h, and plasma collection in SCAD group was first diagnosed and collected within 12 h. The selected people are all from Northeast China with similar living habits and eating styles. The medical history and biochemical indexes such as blood plasma triglycerides, total cholesterol, high and low-density lipoprotein and fasting blood glucose were recorded for each patient. The inclusion criteria for the patients in the AMI (T3) group followed the latest guidelines for the diagnosis of acute myocardial infarction issued by the European Society of Cardiology in 2017 (21). The patients enrolled in the stable coronary artery disease group (SCAD) showed 50–75% stenosis by coronary angiography and met the latest guidelines for the diagnosis of stable coronary artery disease issued by the European Society of Cardiology 2019 (22). The inclusion criteria for the patients in the control (T1) group were no clear vascular lesions confirmed by coronary angiography, neither stenosis nor occlusion of <5% of the main (left trunk, right trunk, etc.) and main branches of the coronary arteries (rotatory branch, anterior descending branch, etc).

The exclusion criteria were based on whether atherosclerosis was caused by secondary factors and immune deficiency diseases. These exclusion criteria were as follows: (1) myocardial infarction associated with percutaneous coronary intervention (PCI) or coronary artery bypass grafting (CABG); (2) type I myocardial infarction, namely secondary myocardial infarction, associated with blood supply imbalance or myocardial infarction caused by elevated catechol levels or coronary artery spasms; (3) myocardial infarction with cardiac surgery or non-cardiac surgery; (4) multiple factors or uncertain myocardial damage caused by uncertain diseases such as severe heart failure, stress cardiomyopathy, severe pulmonary embolism or pulmonary hypertension, septicemia, critical illness, renal failure, stroke, subarachnoid hemorrhage and other severe neurological diseases; (5) immune system diseases and/or hormones use; (6) a history (active or latent) of tuberculosis or evidence of tuberculosis, and (7) chronic infectious disease, recurrent infectious disease or medical history, serious infectious diseases, malignant tumor complications or suspected, confirmed immunodeficiency. We have excluding these factors was due to the fact that there are many secondary causes of atherosclerosis, and numerous variables need to be controlled. Moreover, the direction of the immune deficiency is regularly wide and the influencing factors are numerous, which we assumed unfitting for the current study.

#### Blood Sampling

Fasting peripheral blood (6 ml) of all subjects was extracted in EDTA anticoagulant tubes in the morning and stored at 4°C. Lymphocytes were extracted within 4 h of sample collection, and the reagent used was peripheral blood lymphocyte separation solution (STEMCELL technologies). The detailed steps were as follows: (1) fresh anticoagulant blood was mixed evenly with 0.9% sodium chloride injection of the same volume; (2) the above mixture was carefully added to the equal volume human peripheral blood lymphocyte separation solution and centrifuged at 3,000 r/min for 20 min; (3) after centrifugation, the cells were divided into 4 layers from top to bottom: the plasma layer, the opalescent lymphocyte layer, the clear separation liquid layer and the red blood cell layer. The plasma layer was absorbed and stored in the refrigerator at −80°C.

### Statistical Analysis

The SPSS 22 software was exploited for statistical analysis. The measurement data were normally distributed and X ± S was used for statistical description. The inter-group differences were compared and analyzed by an independent *T*-test. The median and quartile spacing were used for statistical analysis, and the rank-sum test was used for the inter-group differences. The counting data were statistically analyzed by frequency, and the difference between groups was analyzed by x^2^ test.

Screening conditions for differential proteins: |fold change| > 1.2, *p* < 0.05. The first step is to calculate the differential expression of protein between two samples in the comparison group. First, calculate the average value of quantitative value of each sample in multiple repetitions, and then calculate the ratio of the average value between the two samples, which is used as the final differential expression of the comparison group. The second step is to calculate the significance *p*-value of the differential expression of the protein in the two samples. Firstly, the relative quantitative value of each sample is taken as log2 (so that the data conforms to the normal distribution), and then the *P*-value is calculated by the two sample two tailed *t*-test method. When *p* < 0.05, the change of differential expression was more than 1.2 as the change threshold of significant up regulation, and <1/1.2 as the change threshold of significant down regulation.

### Baseline Data of the Selected Patients

A tandem mass tag (TMT) multiplex protein quantitation was further performed and included 10 healthy people, 10 SCAD and 10 AMI while in the PRM validation experiment, 16 healthy subjects were selected, 19 SCAD subjects and 20 AMI subjects. A total number of 30 people were included in TMT and 55 people were included in PRM. In PRM experiment, we expanded the sample size to verify, from 30 in TMT experiment to 55. In the case of large sample size, we observed whether the results of PRM and TMT were consistent. Statistical analysis of the baseline data was conducted using SPSS22. In the baseline data statistics of these patients, no baseline differences were found that significantly influenced the study results. Baseline statistics are shown in Supplementary Tables 1, 2.

### Proteomic Analyses

#### Protein Extraction

Before treatment, peripheral blood was collected and plasma was used. The samples were removed from −80°C and centrifuged at 12,000 g for 10 min at 4°C. Cell debris was removed and the supernatant was transferred to a new centrifuge tube. Pierce™ Top 12, Abundant Protein Depletion Kit (Thermo Company) was used for the removal of high abundance proteins.

#### Liquid Chromatography-Mass Spectrometry

Orbitrap fusion Lumos mass spectrometry. The ion source voltage was set at 2.0 kV, and the peptide parent ions and their secondary fragments were detected and analyzed by high-resolution Orbitrap. The scanning range of primary mass spectrometry is set to 350–1,550 M/Z and the scanning resolution is set to 60,000; The scanning range of secondary mass spectrometry is fixed at 100 m/Z, and the secondary scanning resolution is set at 30,000. The data acquisition mode uses the data dependent scanning (DDA) program, that is, after the primary scanning, select the parent ions of the top 20 peptide segments with the highest signal intensity to enter the HCD collision cell in turn, use 32% fragmentation energy for fragmentation, and also conduct secondary mass spectrometry analysis in turn. In order to improve the effective utilization of mass spectrometry, the automatic gain control (AGC) is set to 5e4, the signal threshold is set to 50,000 ions/s, the maximum implantation time is set to 70 ms, and the dynamic exclusion time of tandem mass spectrometry scanning is set to 30 s to avoid repeated scanning of parent ions.

#### Tandem Mass Tag Mark

Trypsin enzymatic peptides were desalted with Strata X C18 (Phenomenex) and then freeze-dried in a vacuum. The peptides were dissolved with 0.5 M TEAB and marked according to the tandem mass tag (TMT) kit instructions (Thermo). The simple operation was as follows: after thawing, the marked reagent was dissolved in acetonitrile, mixed with the peptide and incubated at room temperature for 2 h. After mixing, the marked peptide was desalted and freeze-dried in a vacuum.

#### Mass Spectrometry Quality Control Detection and Sample Repeatability Test

For biological or technical replicate samples, it was necessary to test whether the quantitative results of biological or technical replicate samples were statistically consistent. Three statistical analysis methods, namely the principal component (PCA), the relative standard deviation (RSD) and the Pearson's Correlation Coefficient analyses were used to evaluate the quantitative repeatability of proteins (as shown in Supplementary Figure 1).

### According to mfuzz Analysis to Find the Relevant Module of Atherosclerosis

Through the cluster analysis of expression patterns, the protein abundance of C1, T2, T3 samples was significantly changed, and the module related to atherosclerosis was found.

### Protein Protein Interaction Network Analysis

The database protein numbers or sequences of differentially expressed proteins screened from different comparison groups were compared with the database of the STRING (v.10.5) protein network, and the differential protein interaction relationship was extracted according to the confidence score >0.7 (high confidence). Then, the R package “networkD3” tool was applied to visualize the differential protein interaction network.

### PRM Validation of Proteomics

The parallel reaction monitoring (PRM) verification included protein extraction, trypsin digestion, LC-MS/MS(Liquid chromatography-tandem mass spectrometry) analysis and data processing with Skyline (v.3.6) software. LC-MS / MS is a tandem mass spectrometry, which can obtain both molecular ion peak and fragment ion peak, so it can be used for qualitative and quantitative analysis.

### Machine Learning-Random Forest Experiment

The target proteins in the three groups were sorted according to their characteristics and then were selected and applied to the ROC analysis. The general rule is that the closer the ROC curve is to the upper left corner, the better the prediction ability of the model is. An intuitive indicator is to use the area under the ROC curve, namely the AUC (area under the curve), in which the AUC value lies between 0.5 and 1, where 0.5 denotes a bad classifier and 1 denotes an excellent one.

## Results

### Overview of Protein Identification

MS was used to identify the proteins in the tested blood probes of the patients grouped in T2, T3 and C1. In total, 10,56,063 secondary spectrograms were obtained by mass spectrometry. The protein theoretical data were analyzed and the number of available effective spectrograms was reduced to 55,833. The utilization rate of spectrograms was 5.3%. Twelve thousand one hundred and twenty five peptides were identified by the spectrogram analysis, among which the specific peptide segment was 11579.0. In our study, 1,147 proteins were identified, 907 of which were quantifiable.

### Target Proteins Were Screened by mfuzz Analysis

**Figure 1:**
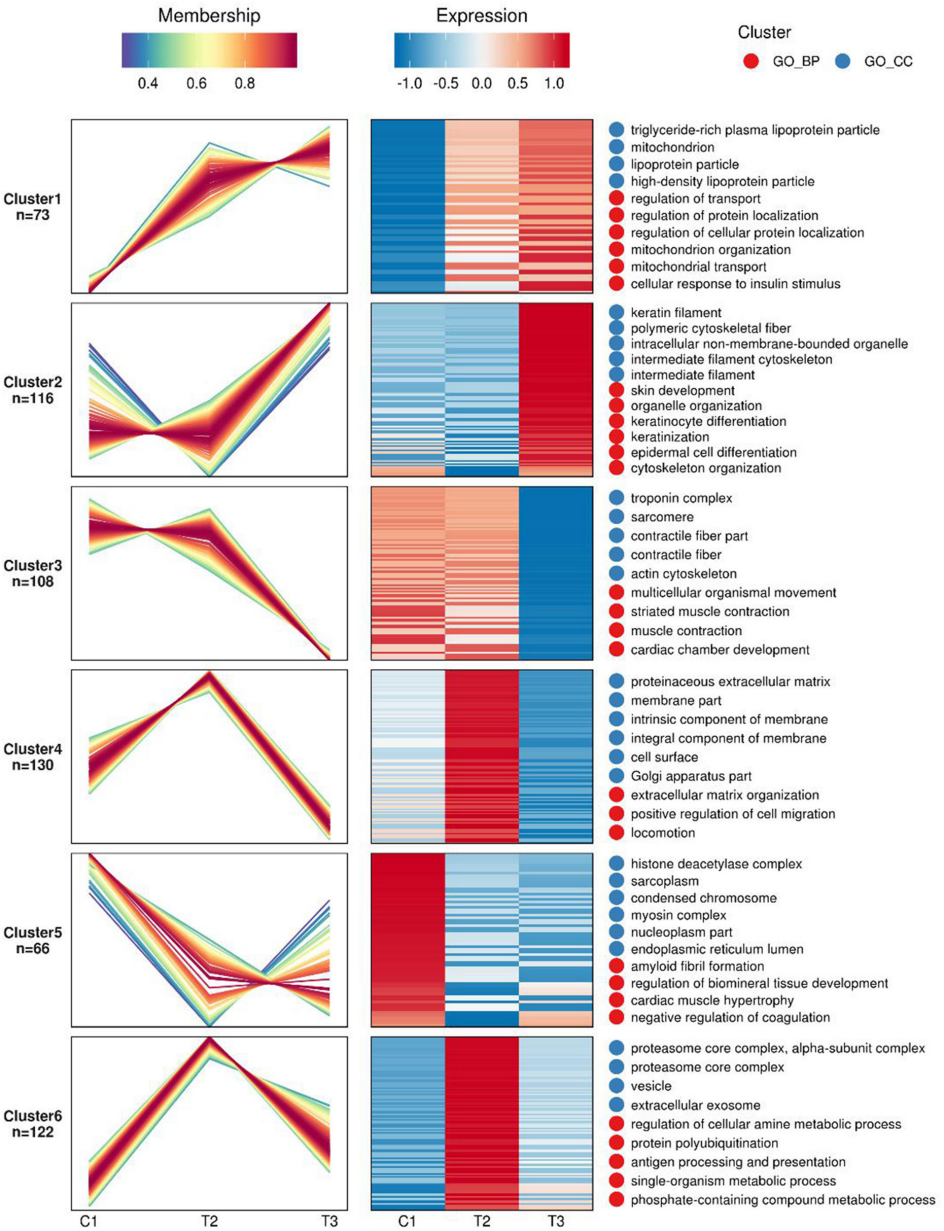
Mfuzz analysis of differential proteins expression in the studied three groups of atherosclerotic patients: The shade of red and blue represents high expression and low expression, respectively.C1 demotes the control group, T2 is the SCAD group, while the T3 is the AMI group.

Gene expression profiles and pathway enrichment analysis to identification of differentially expressed gene and signaling pathways in atherosclerosis was performed through two bioinformatic resources, namely the KEGG (Kyoto Encyclopedia of Genes and Genomes) and the GO (Gene Ontology) with special attention to the factors involved in both lipid metabolism and atherosclerosis. Figure 1 shows the protein heat map, where the ordinate is the protein serial number while the abscissa is the patient's acronym. Through functional analysis, we can see that there are some lipoprotein related functions in the protein function of cluster 1; In cluster 1, there are 5 enrichment function categories highly related to atherosclerosis, of which 3 are the main functions, all of which come from the function of lipid metabolism, namely. Among the protein functions of cluster 3, there are some contraction related functions. The two secondary functions of lipid metabolism are mitochondrial transport and cellular response to insulin stimulus. In cluster 3, there can be two enrichment function categories highly related to atherosclerosis, namely contractual fiber and troponin complex. The specific proteins in the 6 groups are shown in Supplementary Table 3.

### Protein Protein Interaction Network Analysis

**Figure 2:**
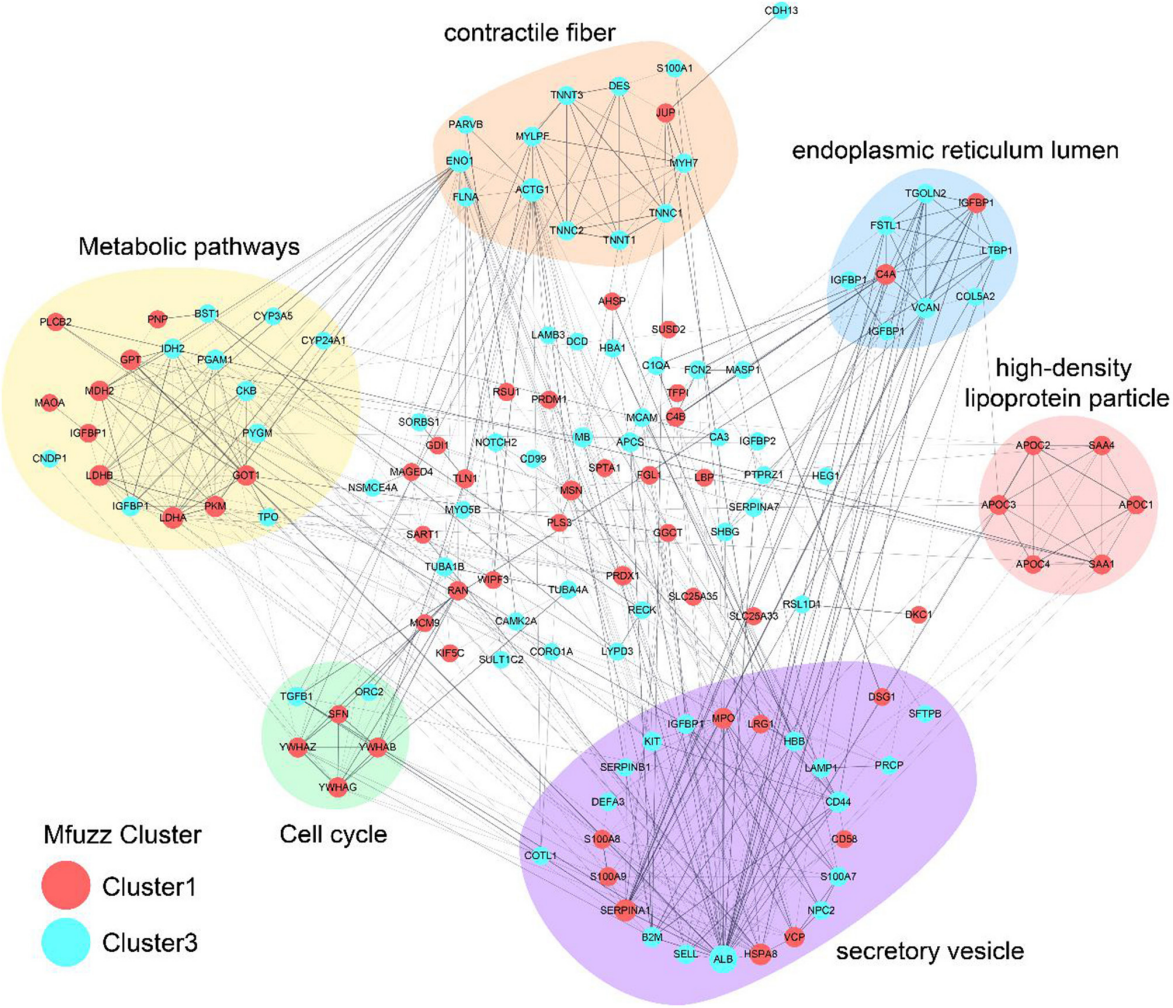
Protein protein interaction network analysis. The lines are interactions, with the proteins in the red circle belonging to Cluster1 and the proteins in the blue circle belonging to Cluster3.

The protein interaction networks of cluster 1 and cluster 3 were analyzed. ALB and SHBG proteins belong to cluster 3. APOC2, APOC3, APOC4 and SAA4 proteins belong to cluster 1 and focus on the function of “high density lipoprotein particle.” See detail in Figure 2.

### Heatmap Results of PRM

**Figure 3:**
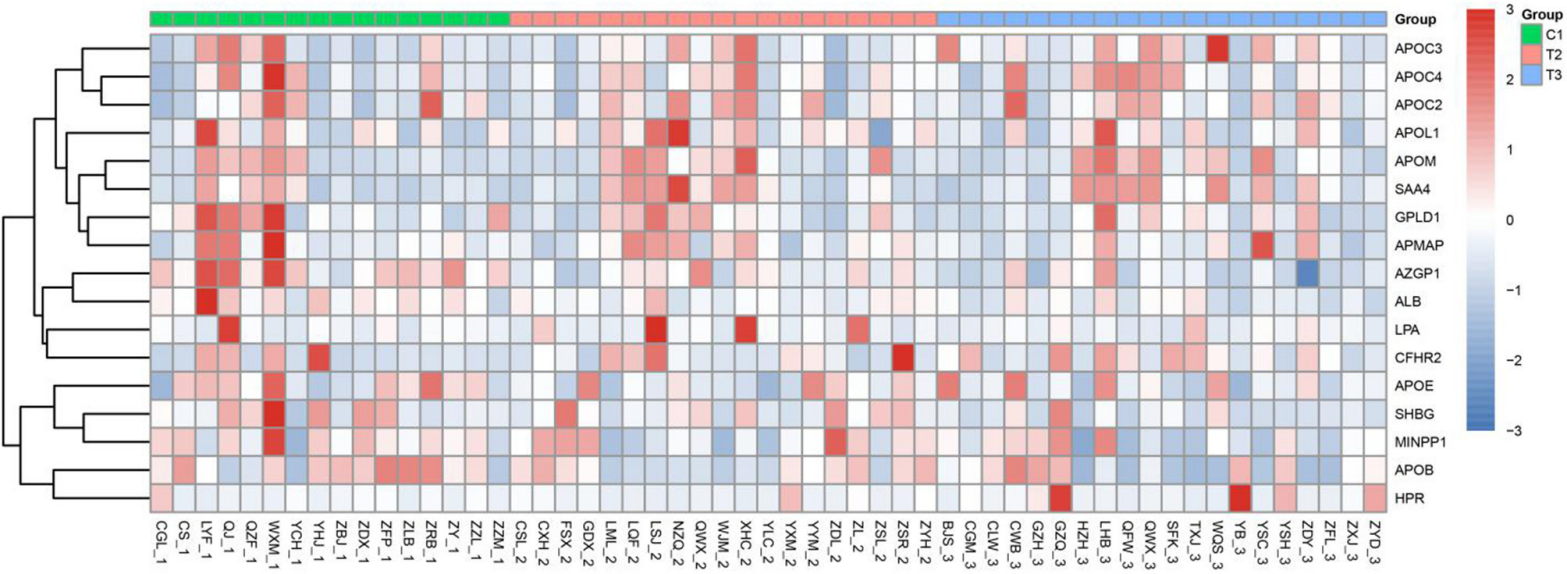
Heatmap results of PRM. The 1, 2, and 3 in the horizontal coordinates represent C1, T2, and T3, respectively. The darker the Red Module, the higher the expression, and the darker the blue module, the lower the expression. The ordinate represents the name of the protein.

During the experimental design, more than 2 unique peptides were used to quantify each protein. PRM was quantified using the peak area. The results of PRM were consistent with those of TMT, and the expression trend of each protein in different groups was consistent with that of protein thermogram (see Figure 3). The distribution diagram of the fragment ion peak area of the selected peptide segment is shown in Supplementary Figure 2. The expression of these proteins increased gradually in C1, T2, and T3 groups. The expression of ALB and SHBG in C1 Group, T2 group, and T3 group decreased gradually. The results of PRM and TMT are consistent.

### Machine Learning-Results of Random Forest Experiment

**Figure 4:**
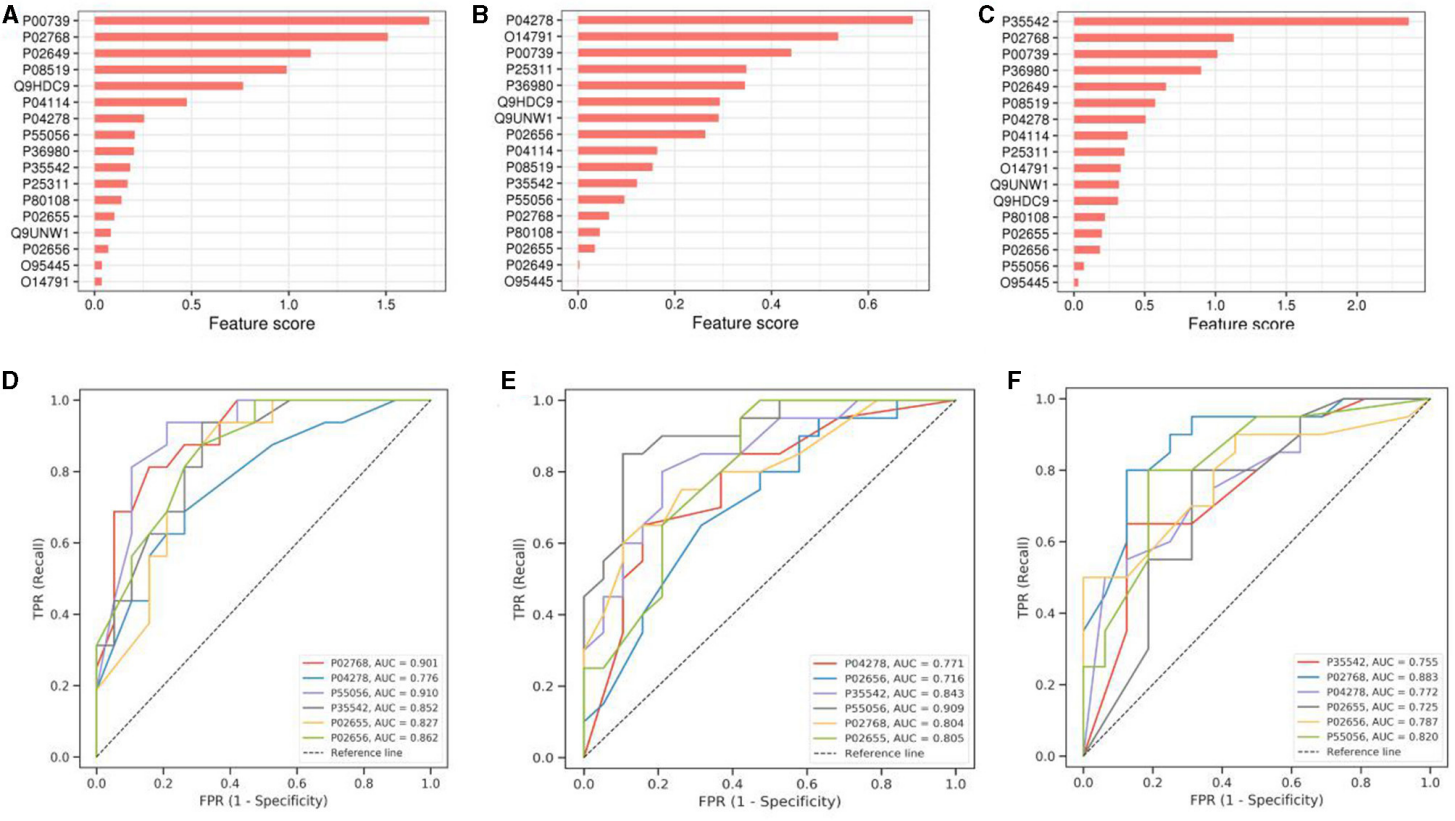
Machine Learning-Results of Random Forest Experiment. **(A–C)** shows the histogram of protein characteristics. **(D–F)** is the ROC curve drawn according to the target protein. SCAD and healthy people are in groups **(A,D)**, SCAD and AMI are in groups **(B,E)**, while the healthy people and AMI are in groups **(C,F)**. The protein name and AUC are in the lower right corner of the corresponding picture. The ordinate is sensitivity and the abscissa is 1-specificit.

To visualize the ranking results of protein expression characteristics, we have drawn the ranking histogram of these characteristics. Figure 4 demonstrates the obtained results. The mRMR feature selection method was used to achieve this goal. In this method, mRMR feature score was calculated for each expression feature by calculating the correlation between expression feature and sample category and the redundancy between the selected expression features.

## Discussion

**Figure 5:**
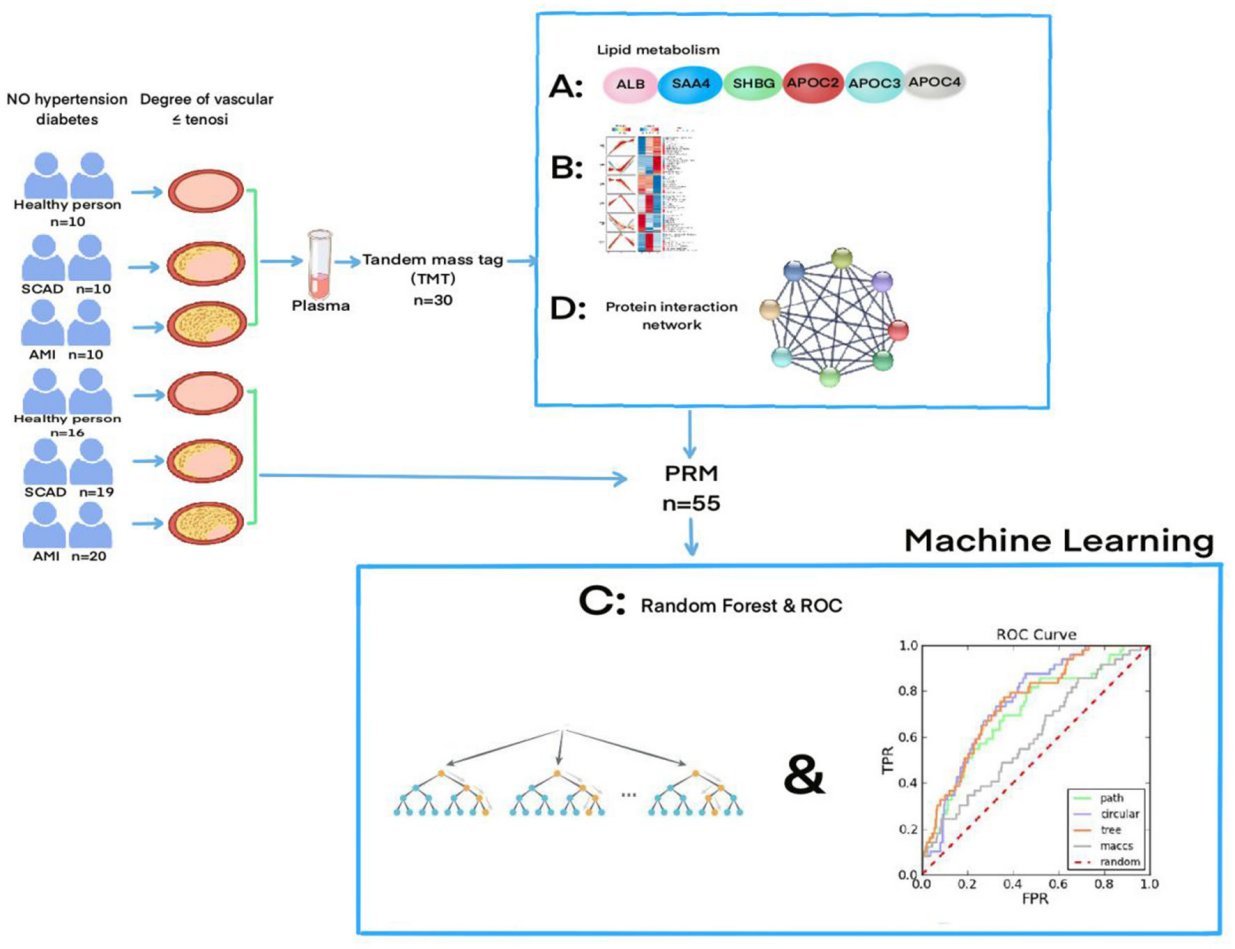
The flowchart of the experiment and the specific experimental process can be found in the “Meterials and Methods part”, and “the result part”.

Atherosclerotic cardiovascular disease is divided into three stages: non-AS (non-Atherosclerotic), SCAD and AMI (23). In this study, 55 patients between 45 and 55 years were selected from whom those with hypertension and type 1 and 2 diabetes were excluded. The main reason for that was the fact that both hypertension and type 1 and type 2 diabetes are recognized as polygenic hereditary diseases with genetic tendency (24). Therefore, the interference of common polygenic diseases should be excluded as far as possible (25). The reason that the age of the included patients was controlled between 45 and 55 years was that most of the SCAD and acute myocardial infarction diseases appear under the age of 45, mainly due to genetic predisposition, while the risk factors over 55 are aging and degeneration of the blood vessels (26). Therefore, we consider that the exclusion of common diseases and the restriction of age more accurately predict that the differential expression of proteins is the key factor in the occurrence of the coronary arteriosclerosis. See Figure 5 for the general experimental flow.

The purpose of the current work is to find the specific protein markers for the diagnosis of different types of coronary atherosclerosis. Through the proteomic analysis of blood plasma of patients diagnosed with atherosclerosis, we have found 1,147 differential proteins, 907, of which were quantifiable (as shown in Supplementary Table 4). Compared with the myocardial tissue, the peripheral blood was easy to obtain, convenient for detection and preservation. Moreover, peripheral blood has been extensively used as an evaluation window for diseases (27–30). Validation of six proteins related to lipid metabolism was performed by expanding sample size. According to the results of TMT, the Mufuzz analysis was used to determine the modules related to arteriosclerosis, the protein interaction network analysis was used, and the PRM was used to verify the results of TMT, finally, the application of machine learning is proved to be correct. The results showed that the expression differences between these six proteins were consistent, and the results were correct and repeatable.

Therefore, we assume that these differences in protein expression can be used as markers for diagnosis and assessment of the severity of coronary atherosclerosis. Atherosclerosis patients are divided into two groups depending on the severity of the condition and they are patients with stable coronary artery disease (SCAD) and acute myocardial infarction (AMI) (31, 32). Both SCAD and AMI are different stages of coronary atherosclerosis and patients experience different symptoms. Those with stable coronary artery disease have pain in the back of the sternum or the front of the heart, or squeezing pain, radiating to the left shoulder and left upper arm. After physical labor, chest tightness, palpitation, shortness of breath occur, which can relieve itself when resting (33). The application of symptoms alone is uncertain, and the combination of symptoms and protein markers will provide necessary help in the early diagnosis of SCAD or AMI (34).

In the PRM validation process, six proteins involved in the lipid metabolism were ALB, APOC2, APOC3, APOC4, SHBG, and SAA4. Interestingly, they do not only participate in the lipid metabolism but also play a special role in the progression of atherosclerosis (35). For example, albumin is the main blood protein and has important physiological functions (36). The concentration of plasma albumin is related to the occurrence and degree of inflammation (37). The process of atherosclerosis is an inflammatory process. Albumin is an important inhibitor of platelet activation and aggregation and induces coronary artery vasoconstriction (38). Several studies conducted at different times showed that low serum albumin level was associated with increased cardiovascular mortality and incidence rate of (39). This is consistent with the fact that the albumin in the AMI group and SCAD group was lower than that in the control group. The second protein Apoc-2 is a modified form of APOC. Similar modifications are apoc-3 and apoc-4. Apoc-2 is the main activator of lipoprotein lipase (LPL), which increases inflammation by promoting macrophages (40). Apolipoprotein C-3 stimulates monocytes, promotes tissue damage (41), and promotes the formation and outcome of alcoholic cirrhosis. Besides, it increases the risk of Alzheimer's disease by increasing high-density lipoprotein (HDL) (42, 43). Apolipoprotein C-4 increases the risk of diabetes in young people and affects the lipid profile of patients (44). Apoc-4 may affect the development of hepatitis B together with other members of the APOC family (45).

Endothelial dysfunction, on the other hand, is an important criterion for prediction of the prevalence of the cardiovascular disease in men and premenopausal women (46). Sex hormones and sex hormone-binding globulin (SHBG) are potential regulators of male endothelial function, and SHBG is positively correlated with endothelial function (47). This is consistent with our conclusions. SHBG in T2/C1, T3/C1, T3/T2 (SCAD group/control group, AMI group/control group, AMI group/SCAD group) showed a trend of low expression and further confirmed that SHBG was negatively correlated with the atherosclerosis type.

The sixth protein SAA4 is a member of serum amyloid A (SAA). So far, four SAA subtypes have been identified, which are closely related to lipid metabolism in tissues and serum, and play a role in promoting inflammation (48). SAA4 is a major acute phase reactant expressed in coronary artery macrophages, which suggests that SAA4 plays a special role in inflammatory processes including atherosclerosis (49).

In conclusion, our study is the first to reveal the changes of plasma proteins between different types of atherosclerosis. Six of these proteins were found to promote the development of atherosclerosis by participating in the lipid metabolism pathway. Changes in plasma proteins contribute to the identification of their potential as biomarkers and provide a new method for the clinical diagnosis and treatment of atherosclerosis (50, 51). More importantly, the establishment of effective and safe personalized treatment programs can meet the needs of the development of modern precision medicine and personalized diagnosis and treatment. The relationship between differentially expressed proteins and the exact molecular mechanisms of action (e.g., transcriptional and post-translational modifications) of atherosclerosis needs to be validated by further studies.

## Data Availability Statement

The datasets presented in this study can be found in online repositories. The names of the repository/repositories and accession number(s) can be found at: ProteomeXchange [accession: PXD028664].

## Ethics Statement

This study was approved by the Ethics Committee of China Japan United Hospital of Jilin University. The patients/participants provided their written informed consent to participate in this study.

## Author Contributions

PY and FM: conceptualization. HM: data curation, investigation, software, and writing–original draft. KS: formal analysis. FM: funding acquisition and project administration. HM and FM: methodology. XL: resources. ZY: supervision. HM and JR: validation. JR and YC: visualization. PY: writing–review and editing. All authors contributed to the article and approved the submitted version.

## Funding

This research was funded by the Health Commission of Jilin Province in 2018 of Fanbo Meng, grant number: 2018SCZ008.

## Conflict of Interest

The authors declare that the research was conducted in the absence of any commercial or financial relationships that could be construed as a potential conflict of interest.

## Publisher's Note

All claims expressed in this article are solely those of the authors and do not necessarily represent those of their affiliated organizations, or those of the publisher, the editors and the reviewers. Any product that may be evaluated in this article, or claim that may be made by its manufacturer, is not guaranteed or endorsed by the publisher.

## Supplementary Material

Supplementary Figure 1 Sample repeatability test.

Supplementary Figure 2 The ion peak area distribution of peptide fragment.

Supplementary Table 1 Clinical and laboratory characteristics of the experimental group.

Supplementary Table 2 Clinical and laboratory characteristics of the validation group.

Supplementary Table 3 Cluster 1–6 proteins.

Supplementary Table 4 Identified_information.
