# Supplementary material for: Investigation of Specific Proteins Related to Different Types of Coronary Atherosclerosis
Source: Front Cardiovasc Med. 2021 Oct 22;8:758035. doi: 10.3389/fcvm.2021.758035 (PMC8569131; doi:10.3389/fcvm.2021.758035)
Supplement: Supplementary Figure 1 — Sample repeatability test. [file Data_Sheet_1.DOCX]

**
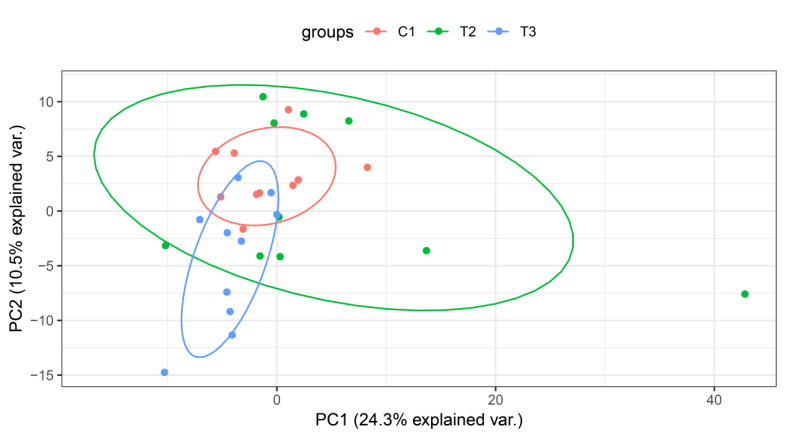
**

**Fig.S1a**

Fig.S1a shows the protein quantitative principal component analysis results of all samples. In fig.S1a, the better the aggregation degree between repeated samples means the better the quantitative repeatability (C1 was the control group, T2 was the stable coronary heart disease group, and T3 was the acute myocardial infarction group).

**
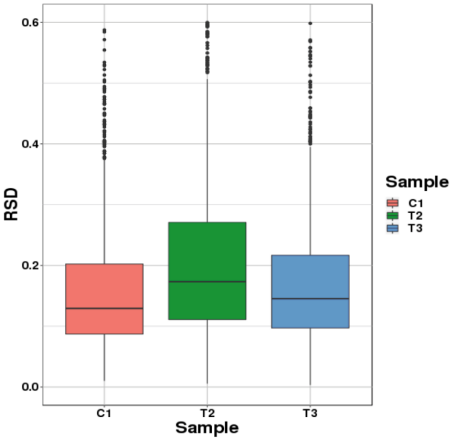
**

**Fig.S1b**

Fig.S1b is a boxplot drawn with the relative standard deviation (RSD) of the quantitative protein values among the repeated samples. The smaller the overall RSD value is, the better the quantitative repeatability will be

**
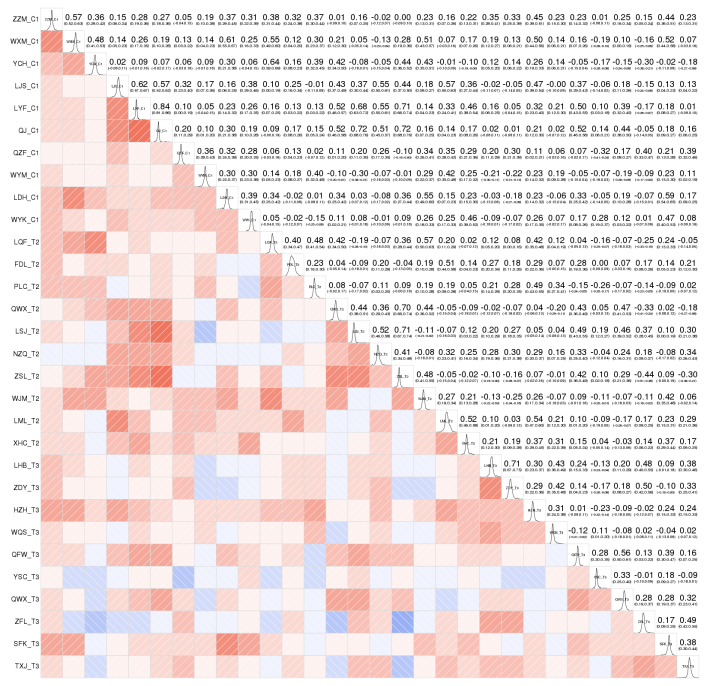
**

**Fig.S1c**

Fig.S1c is a heat map for calculating the Pearson correlation coefficient between all sample pairs. This coefficient is a value that measures the degree of linear correlation between the two sets of data. When the Pearson coefficient is closer to -1, it is a negative correlation, the closer to 1 is a positive correlation, and the closer to 0 is no correlation.
