# Supplementary figures and images for: Investigation of Specific Proteins Related to Different Types of Coronary Atherosclerosis
Source: Front Cardiovasc Med. 2021 Oct 22;8:758035. doi: 10.3389/fcvm.2021.758035 (PMC8569131; doi:10.3389/fcvm.2021.758035)

The ion peak area distribution  
of peptide fragment \_55Samples

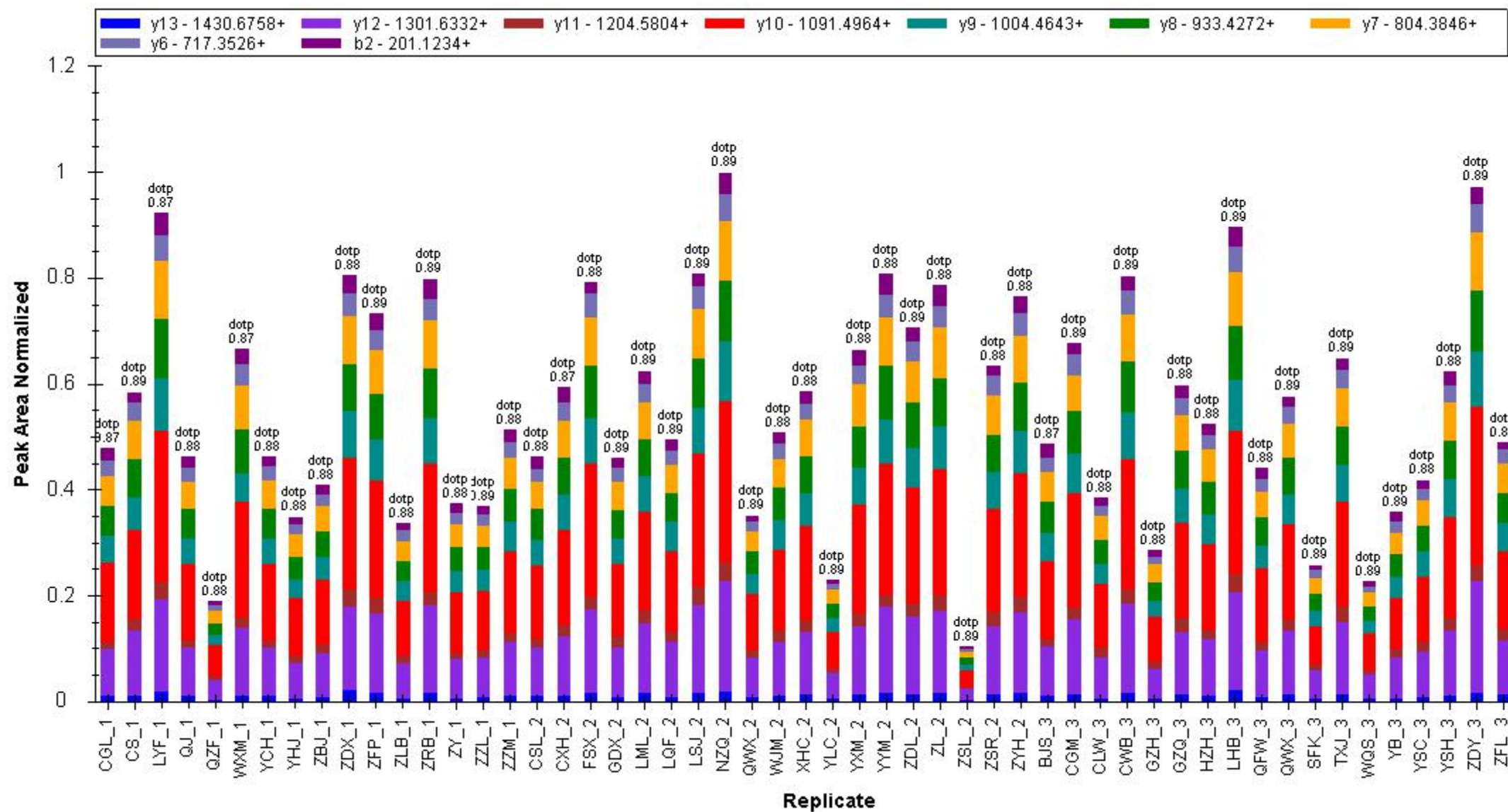

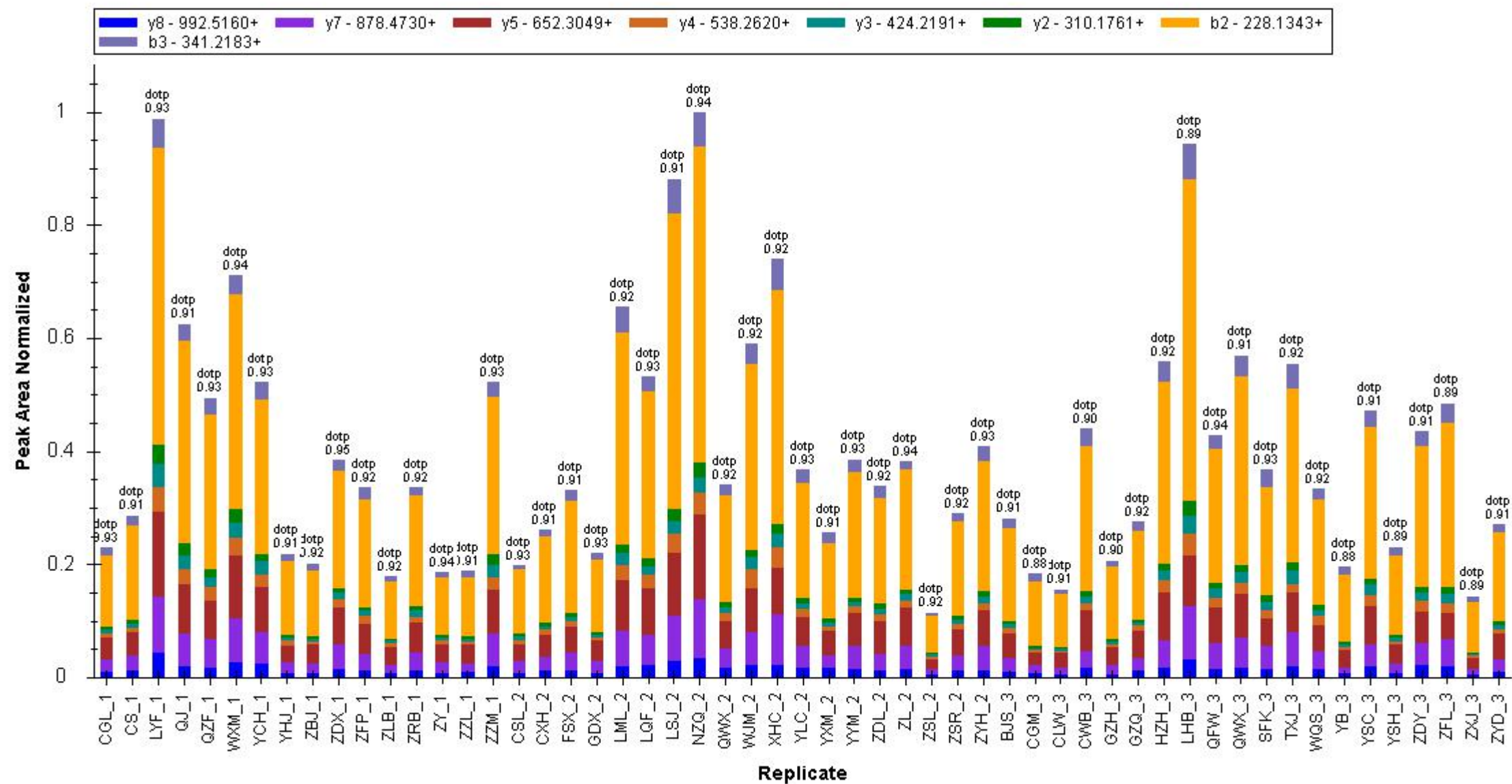

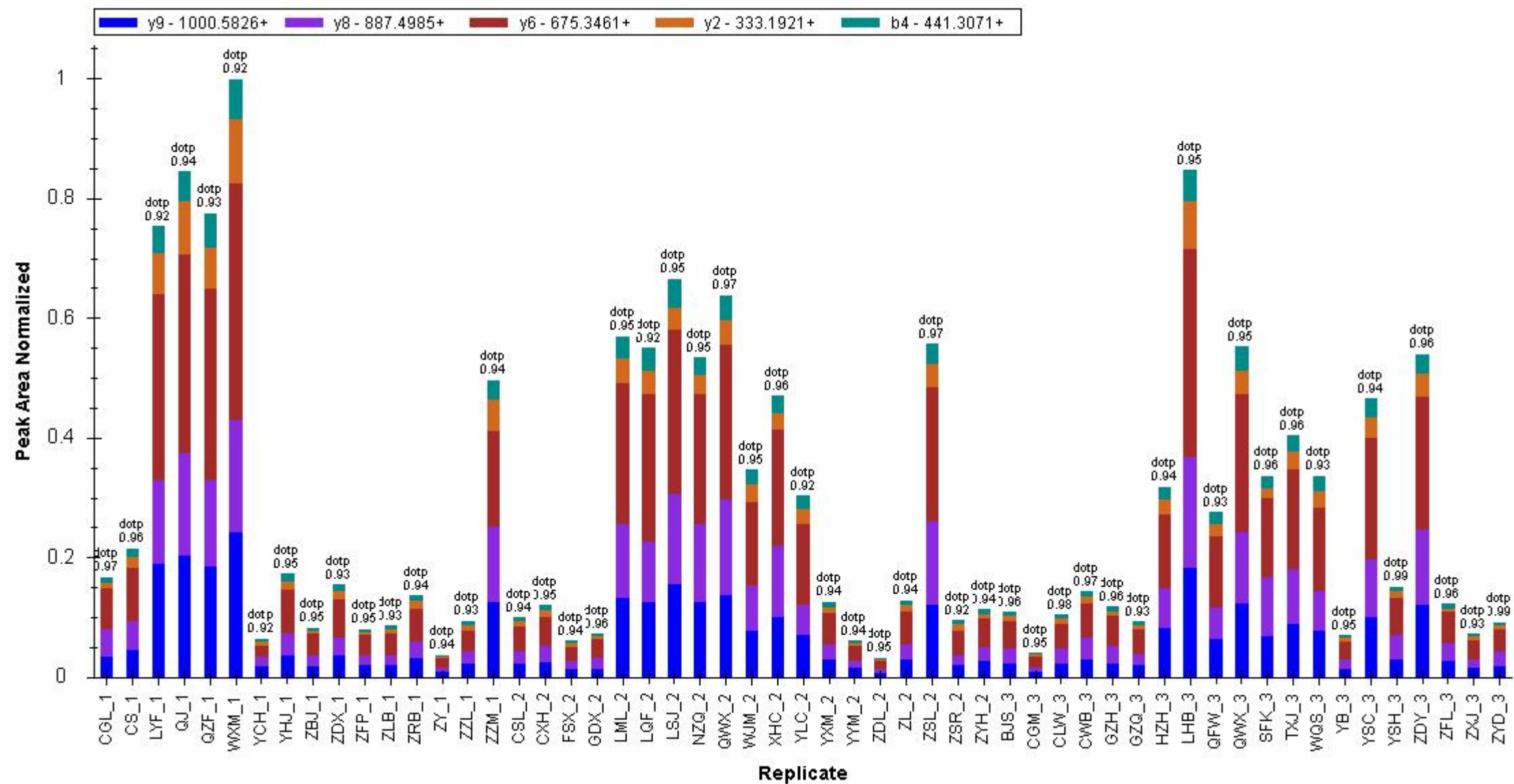

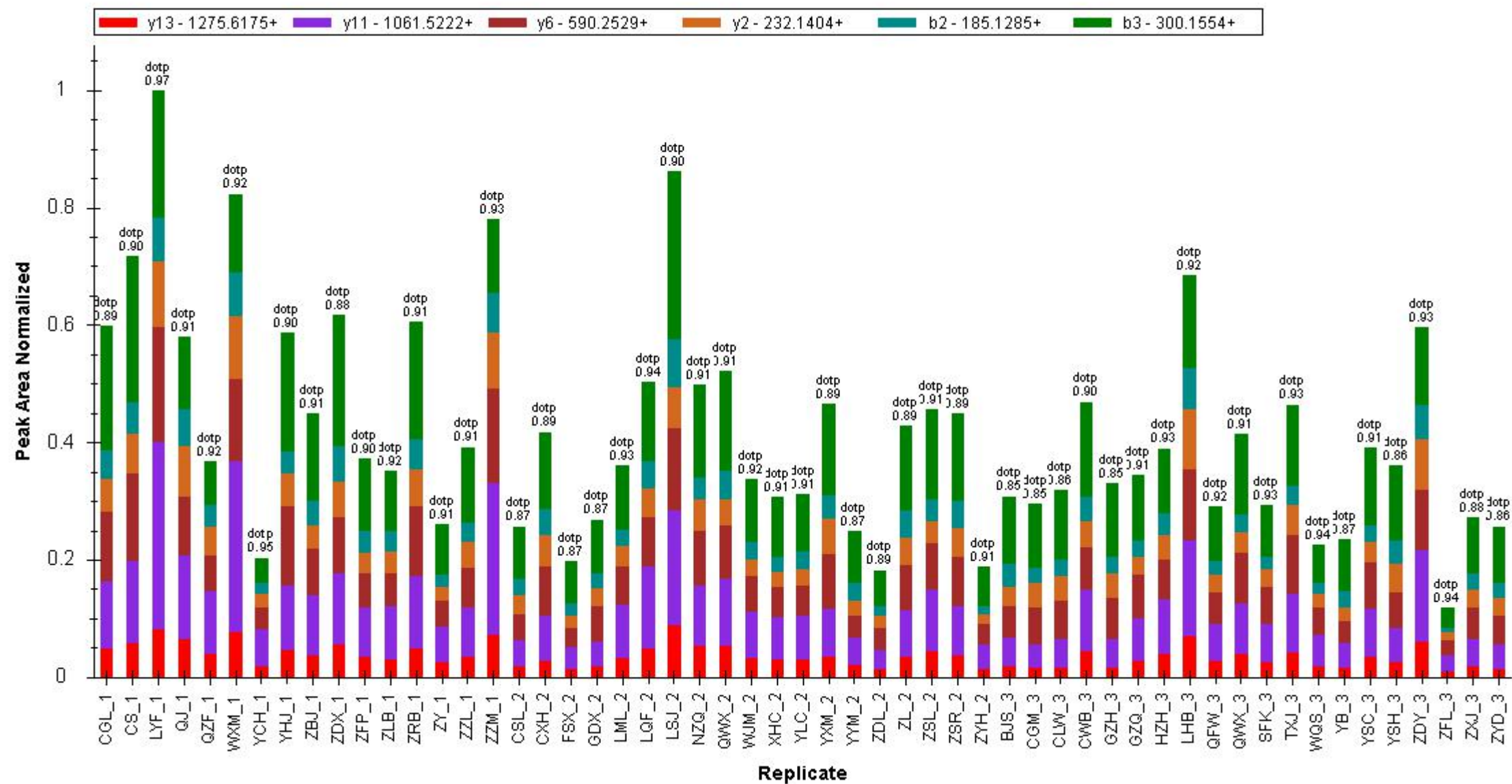

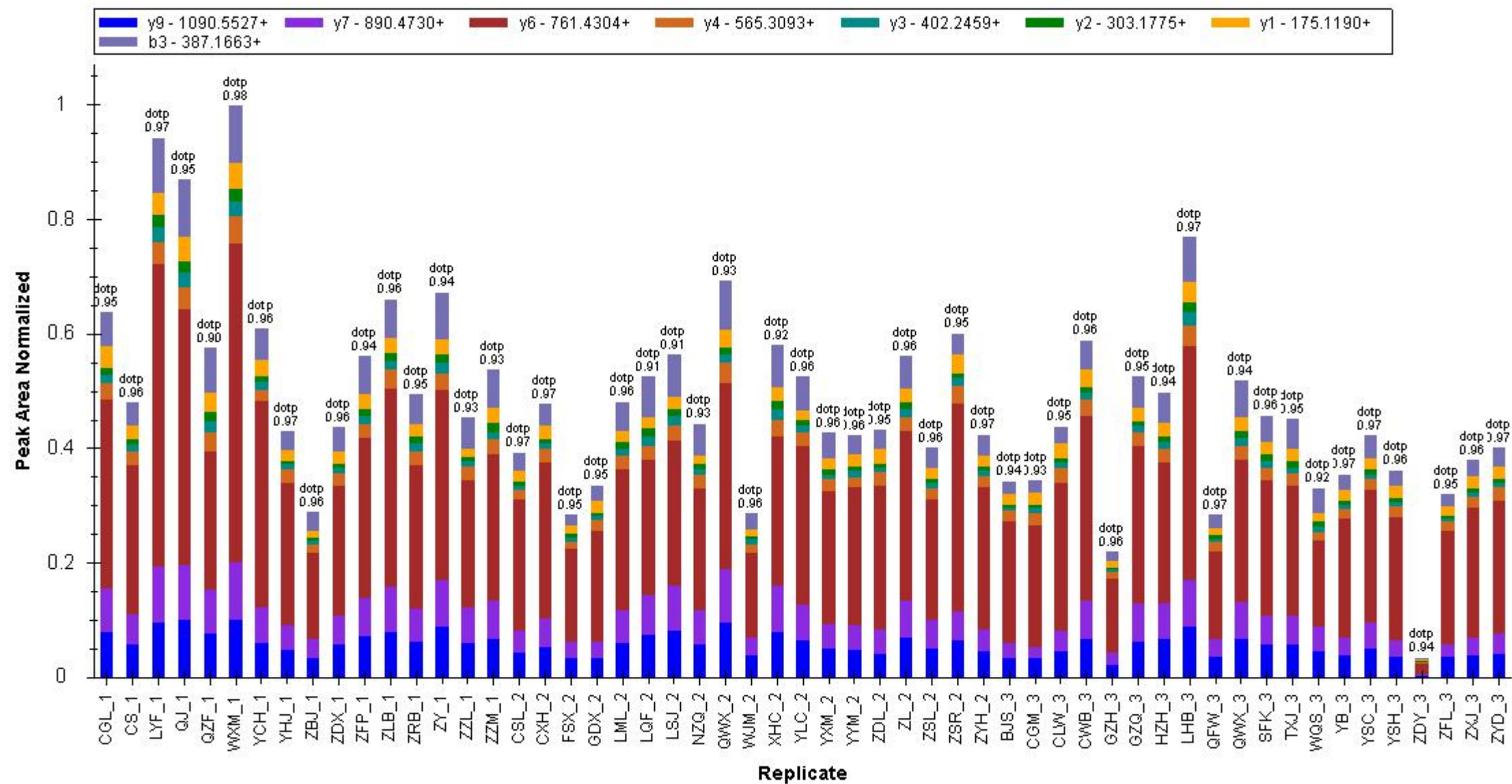

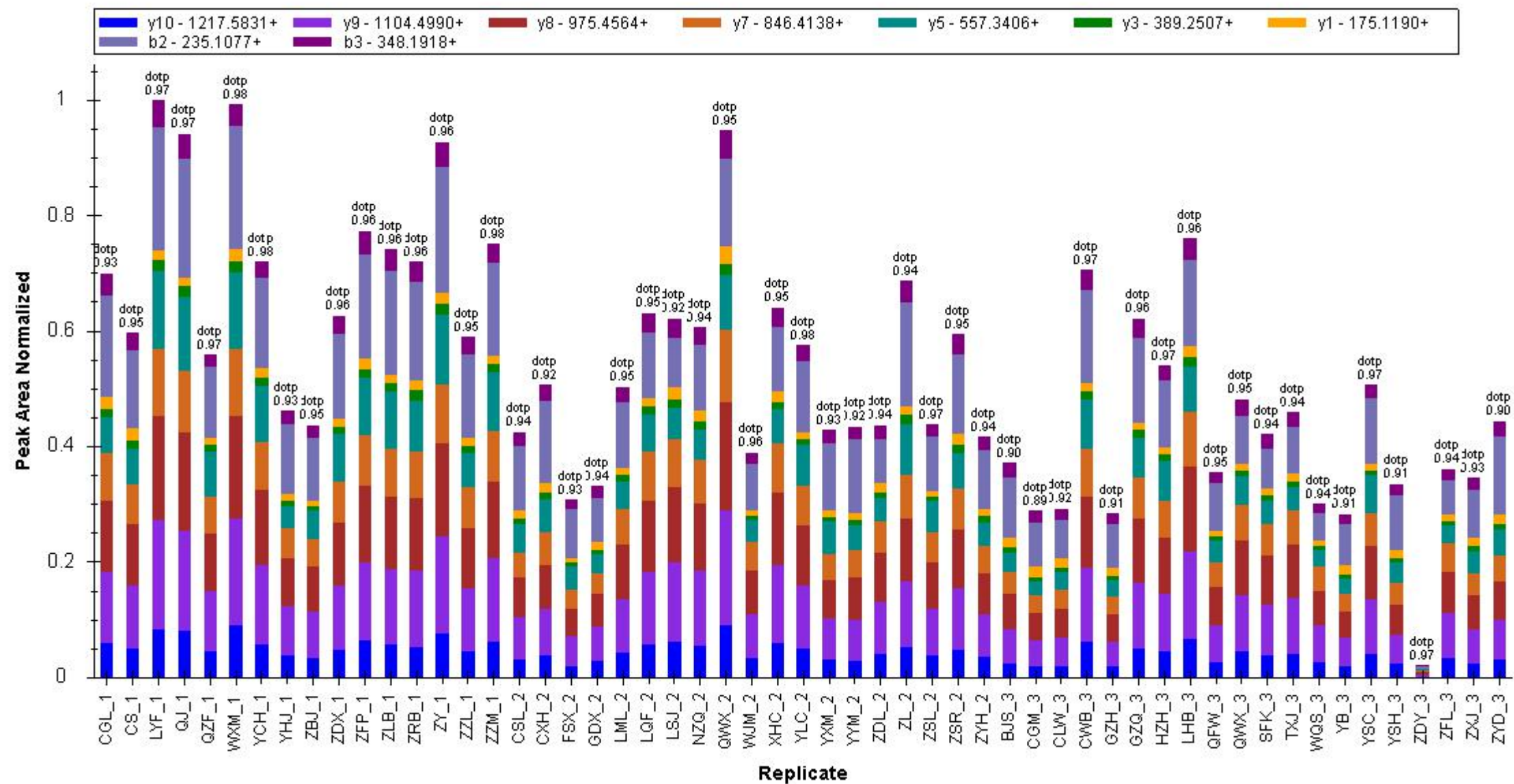

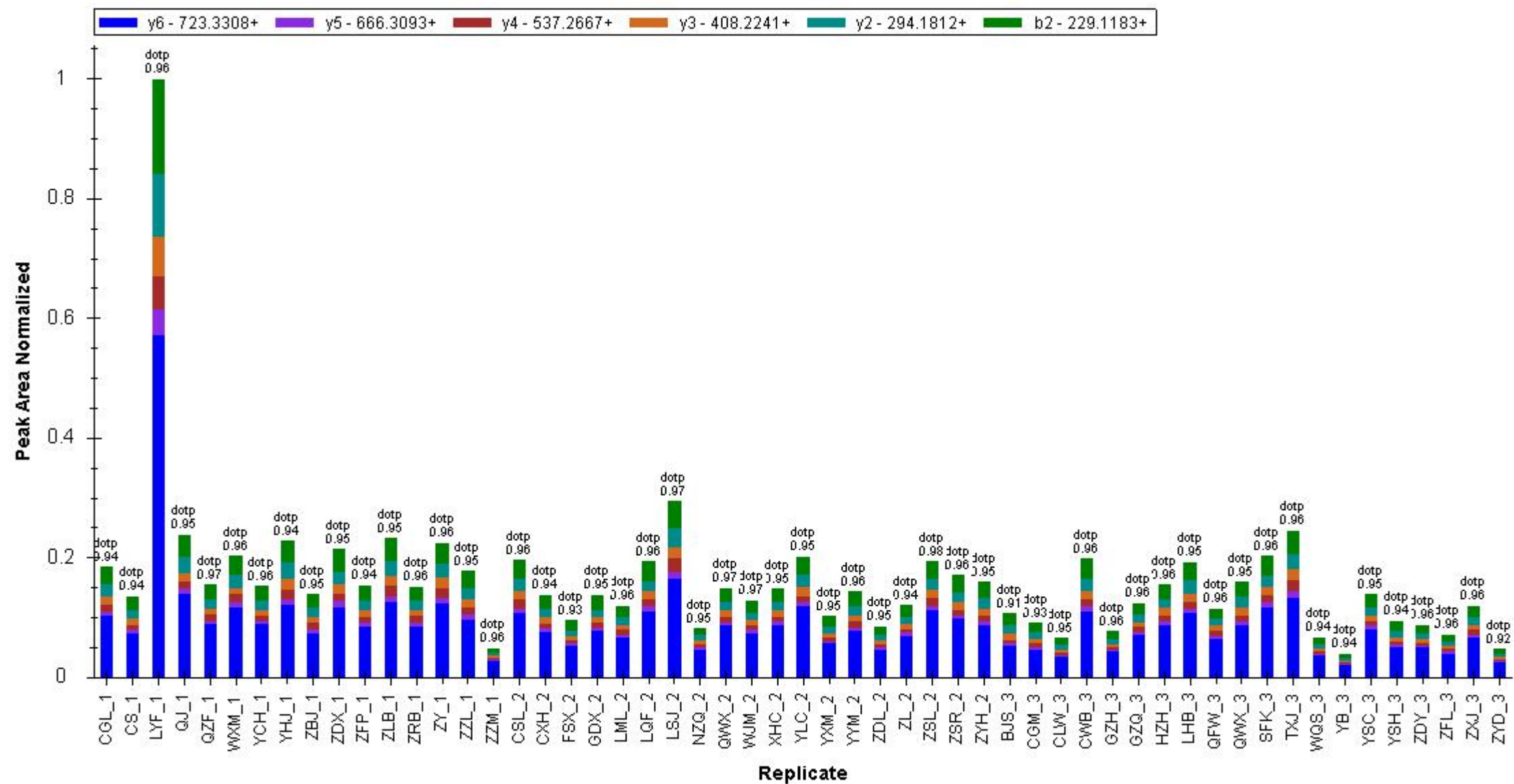

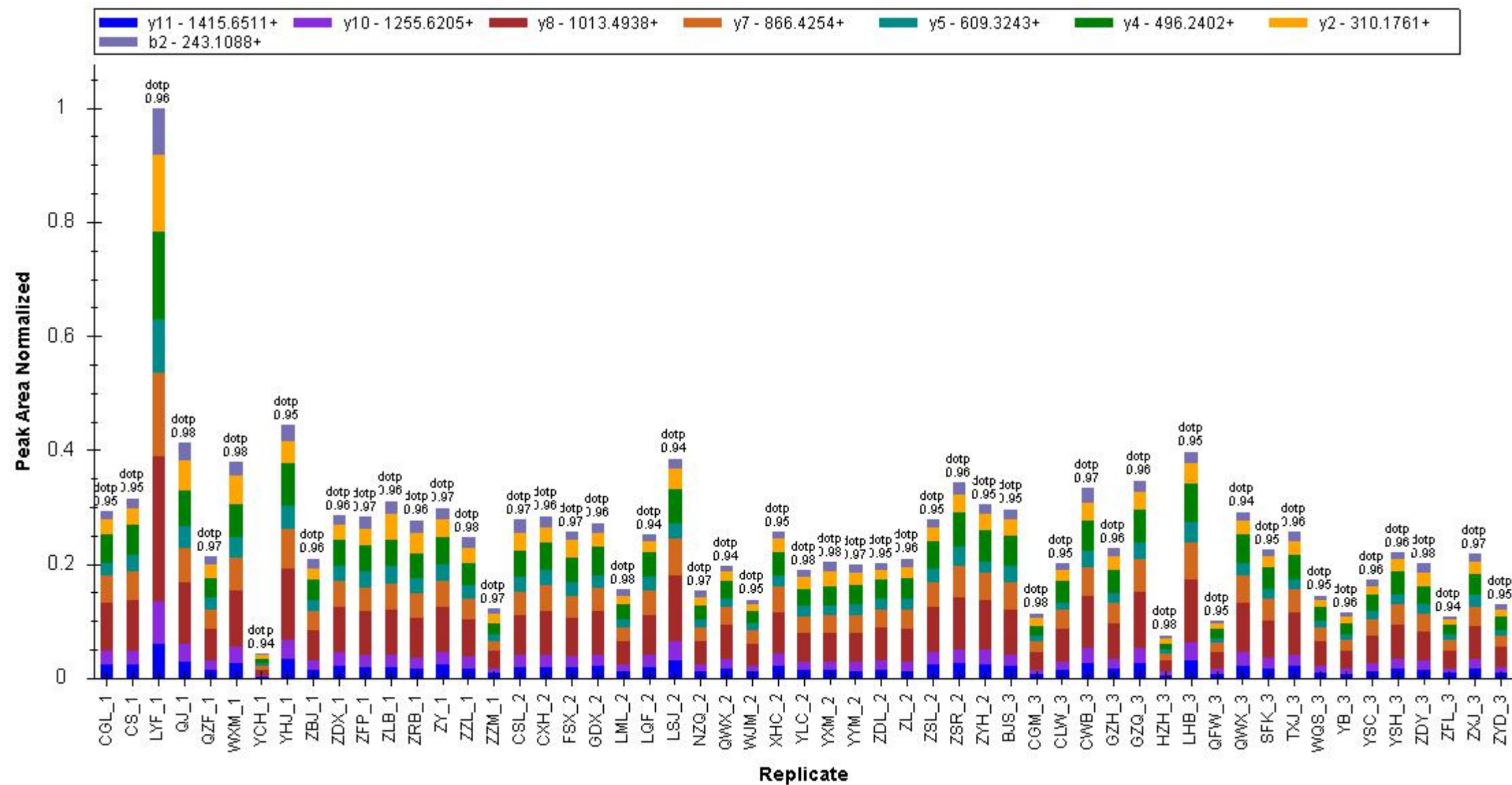

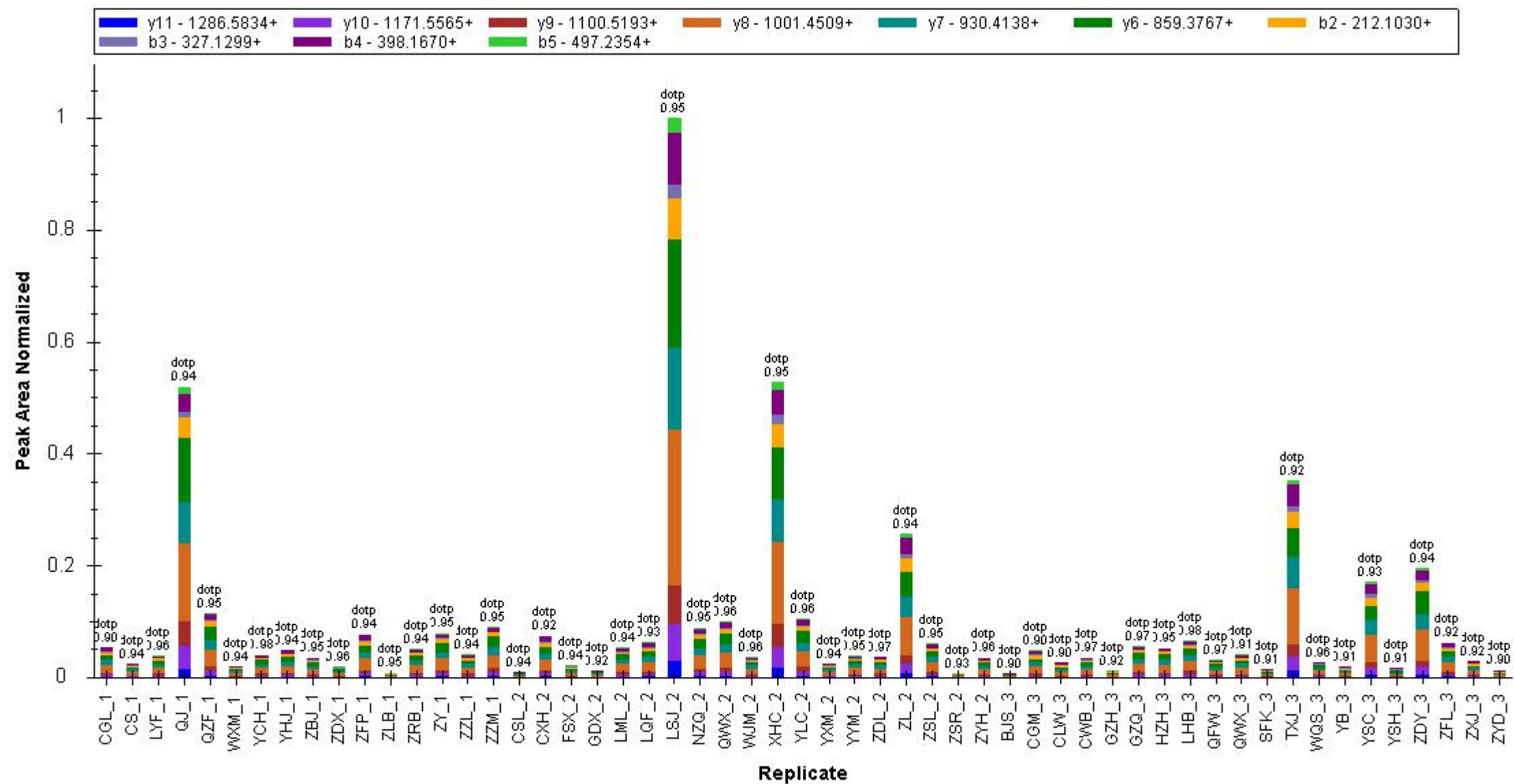

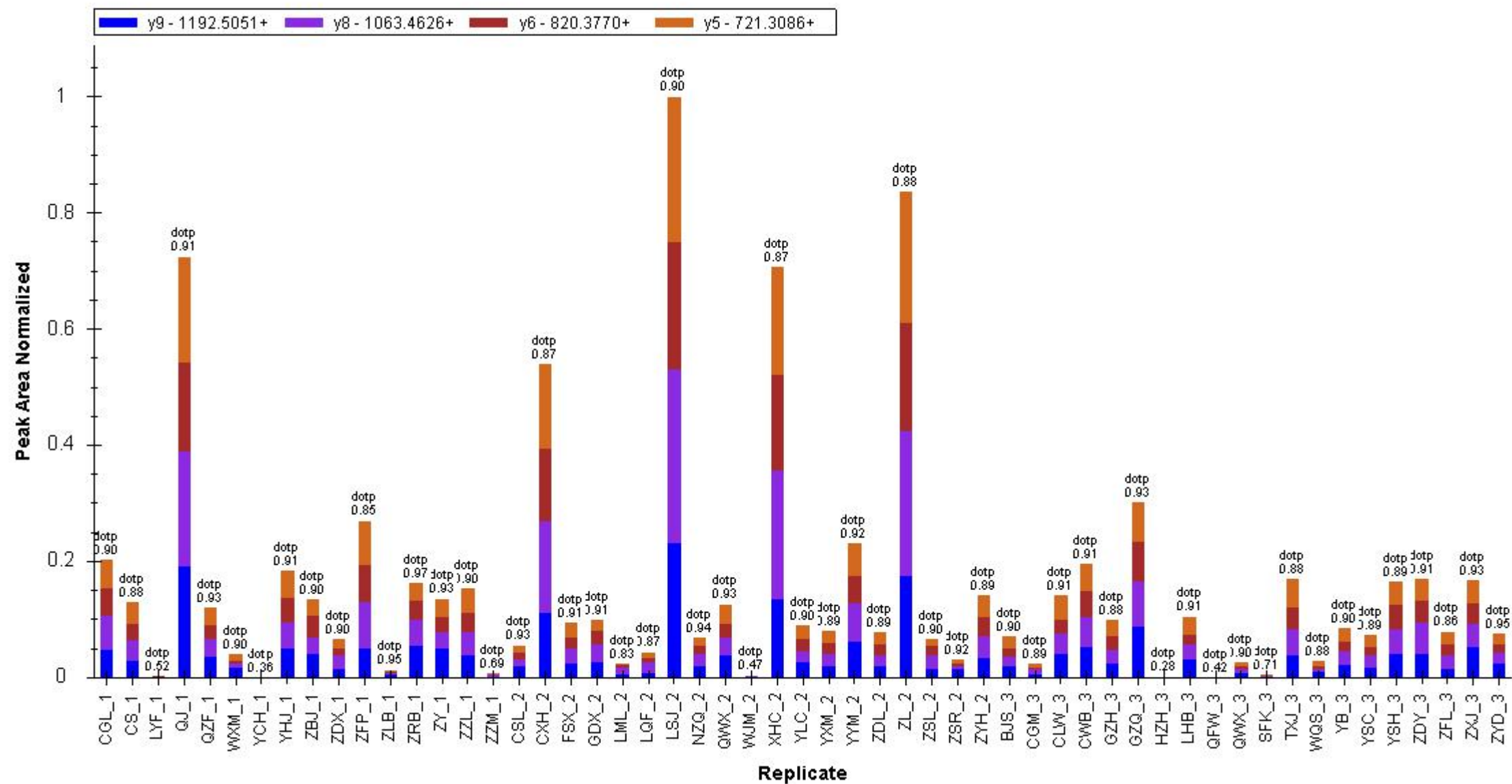

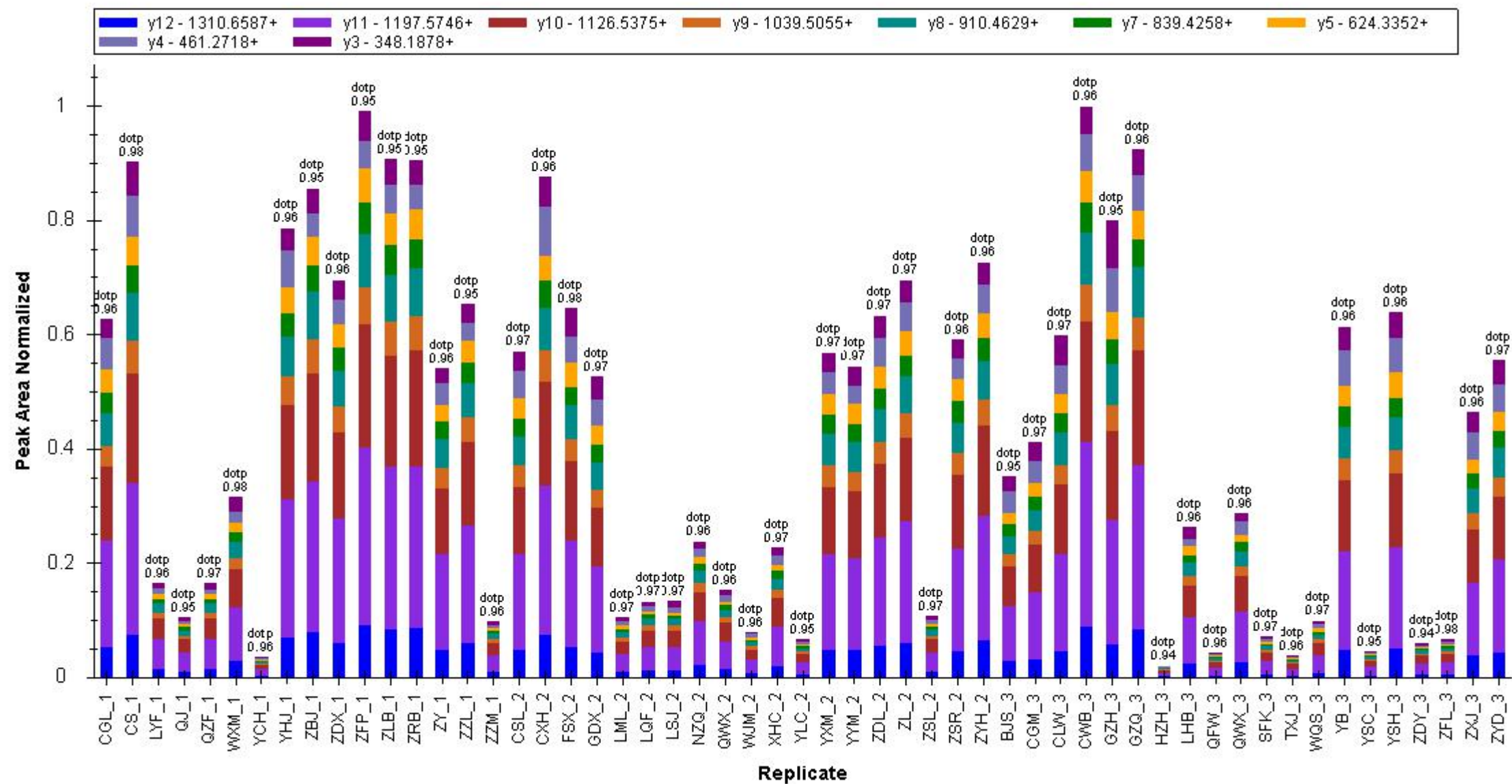

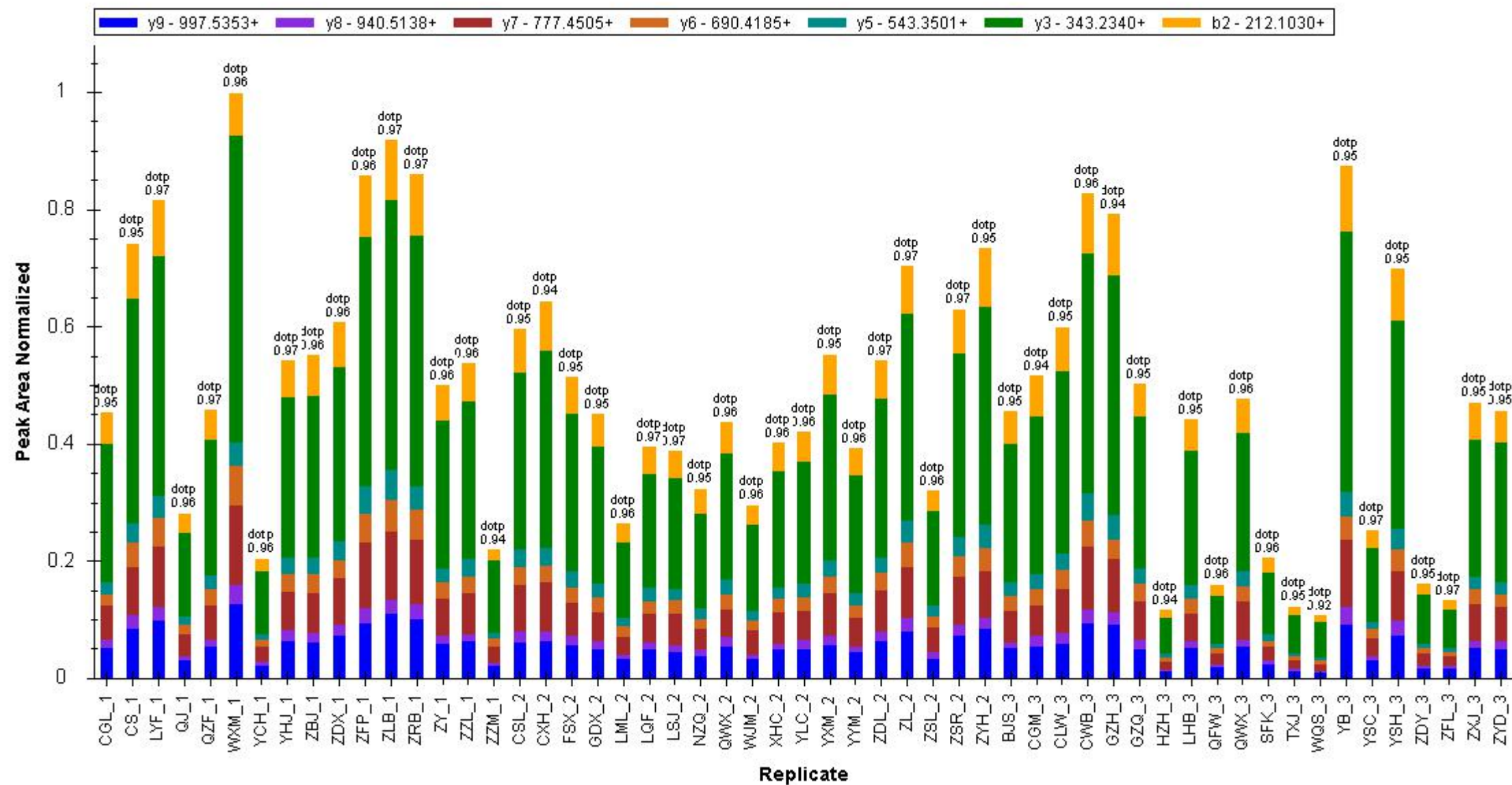

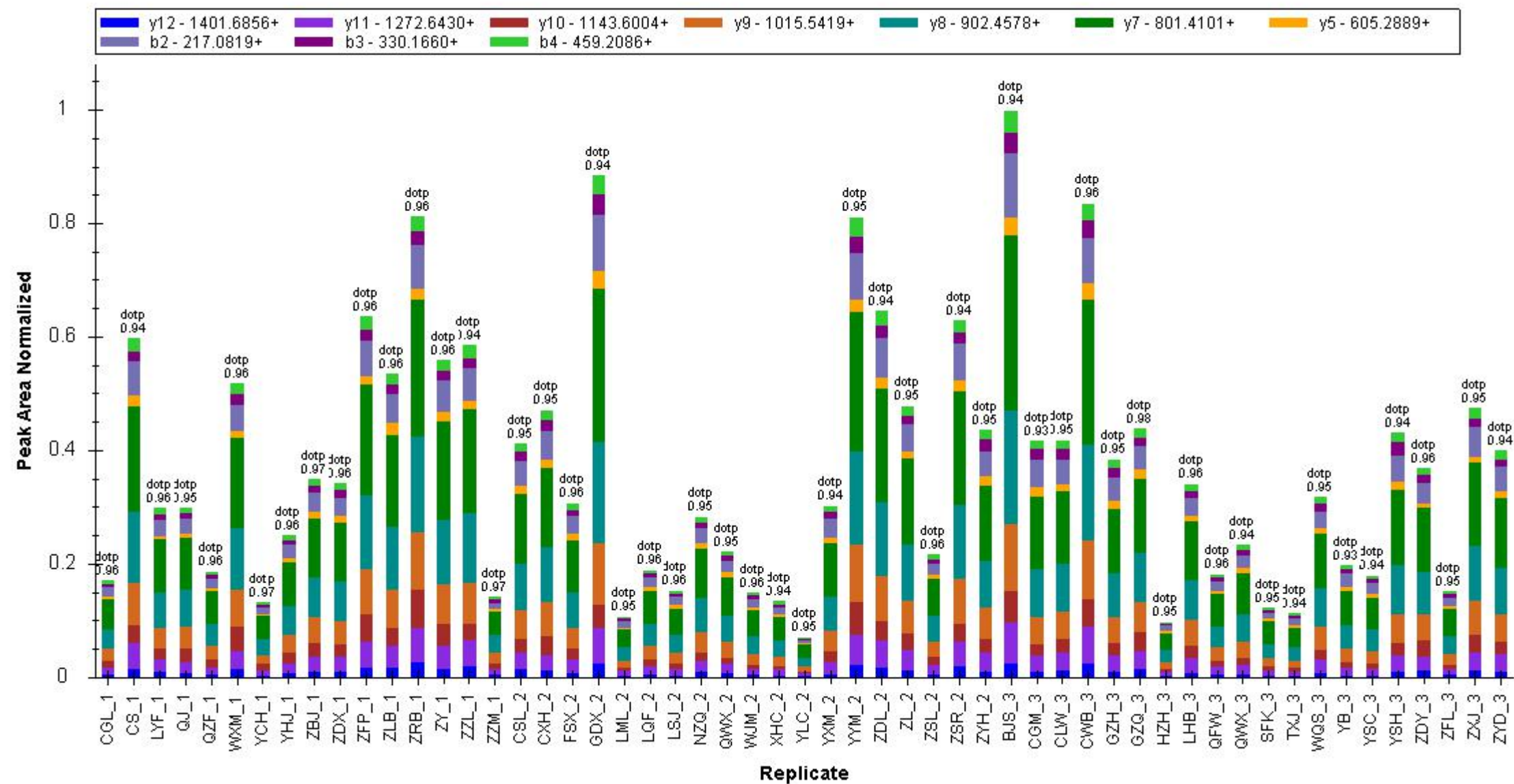

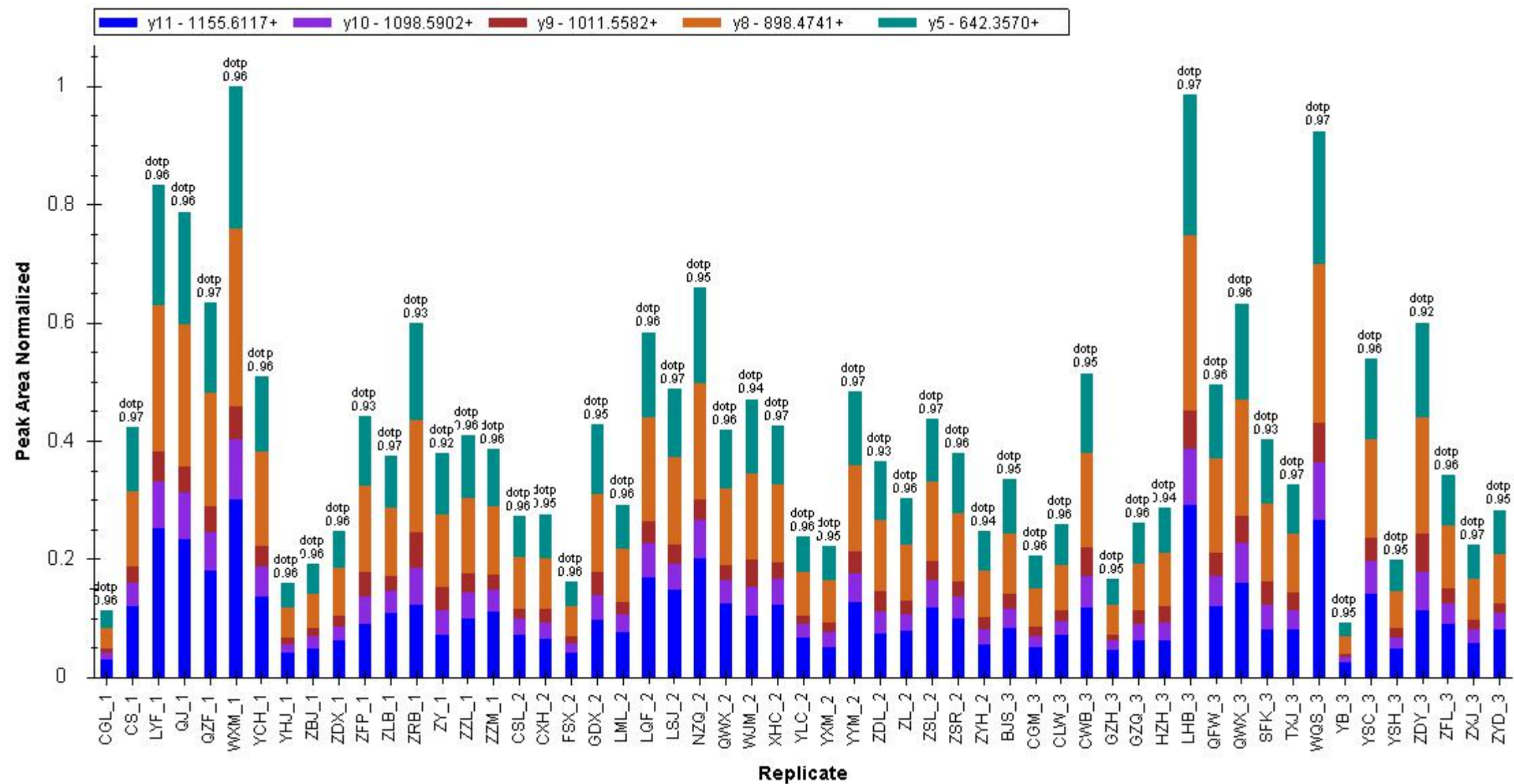

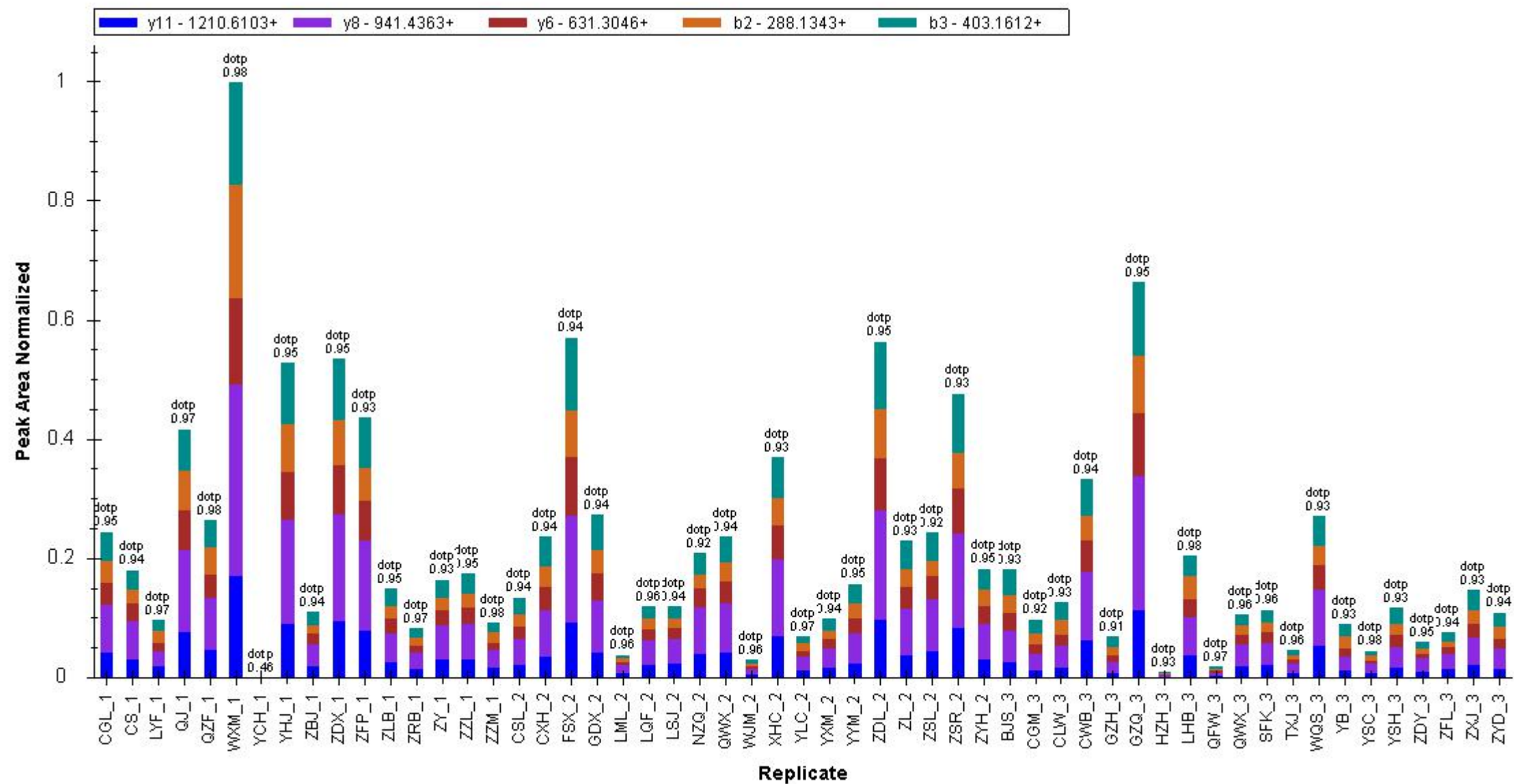

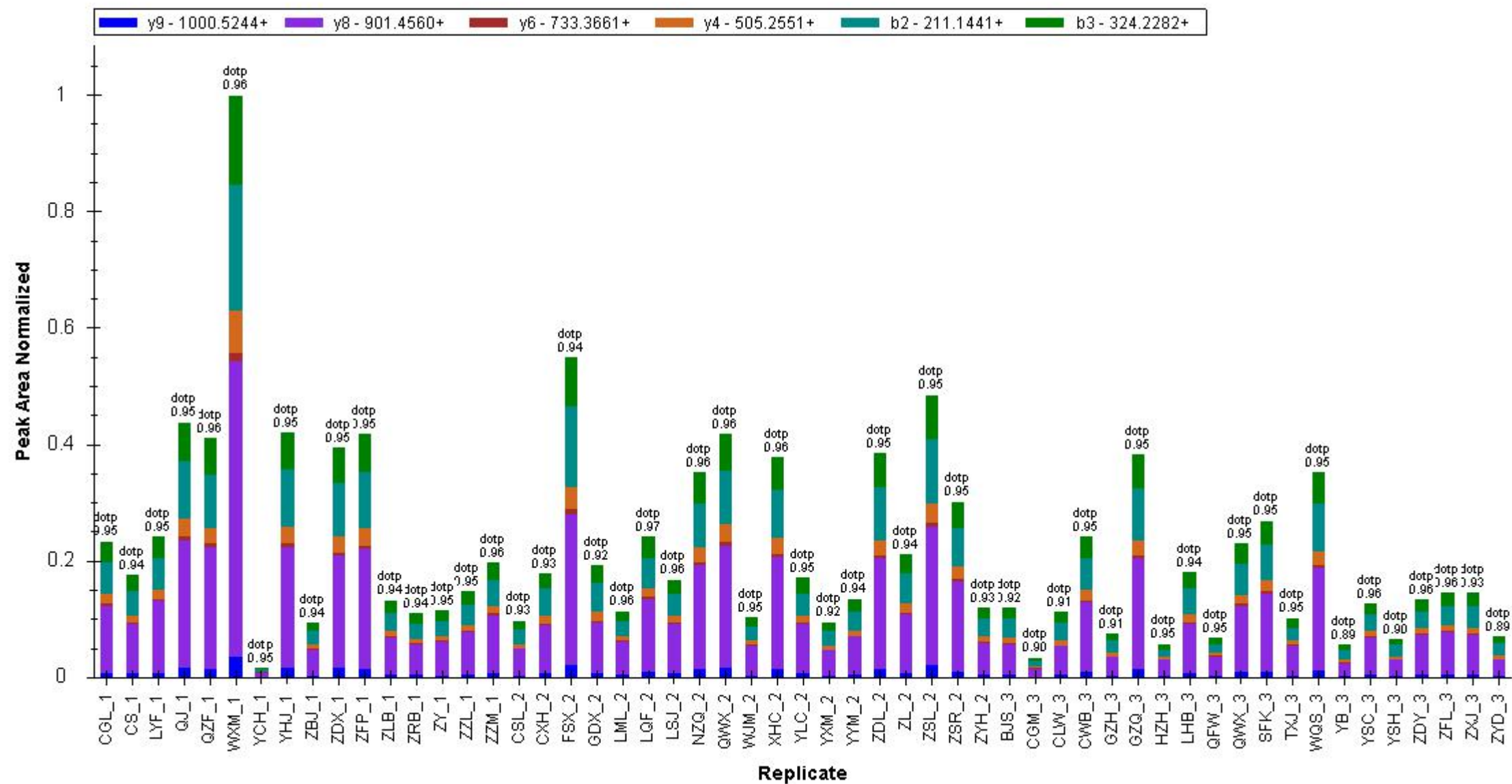

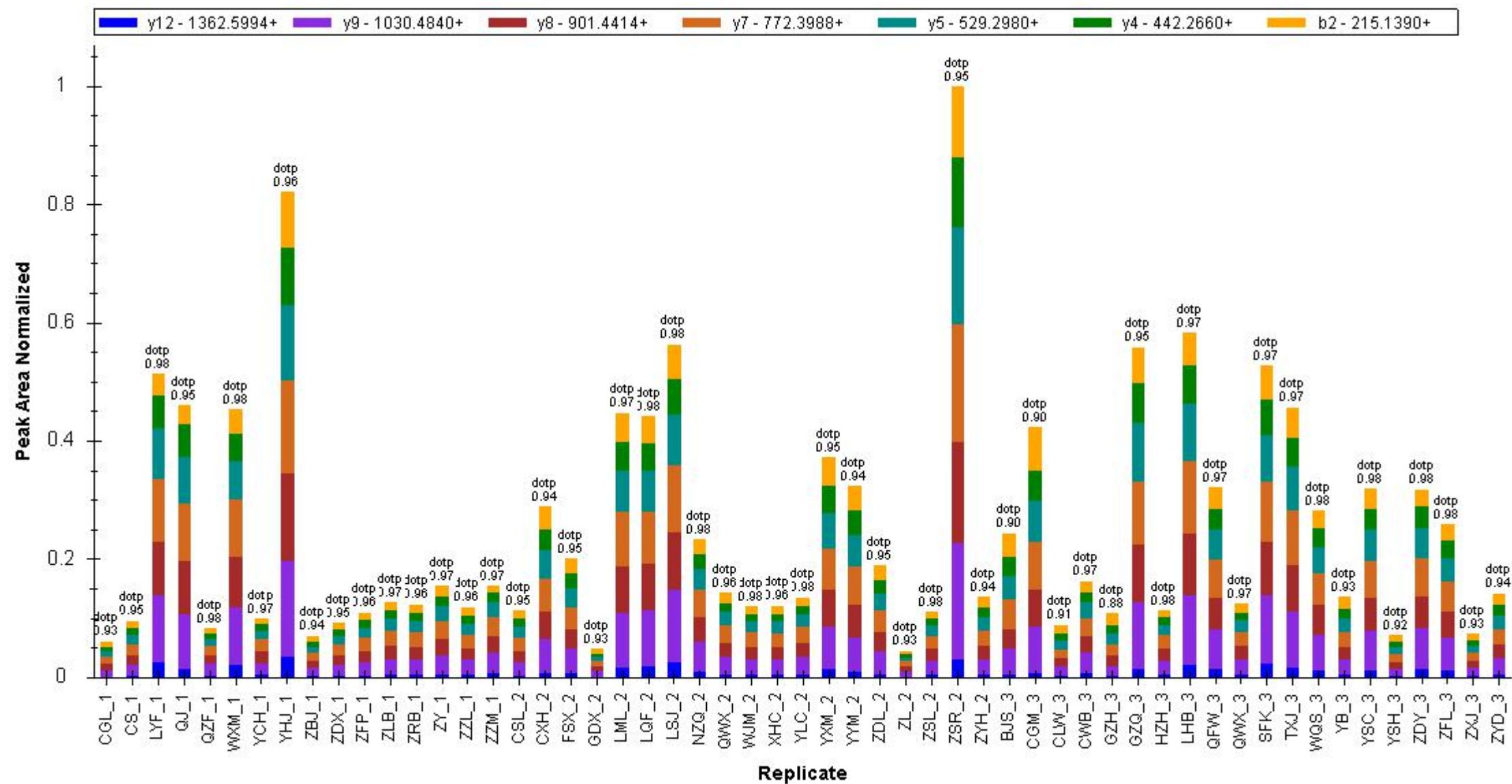

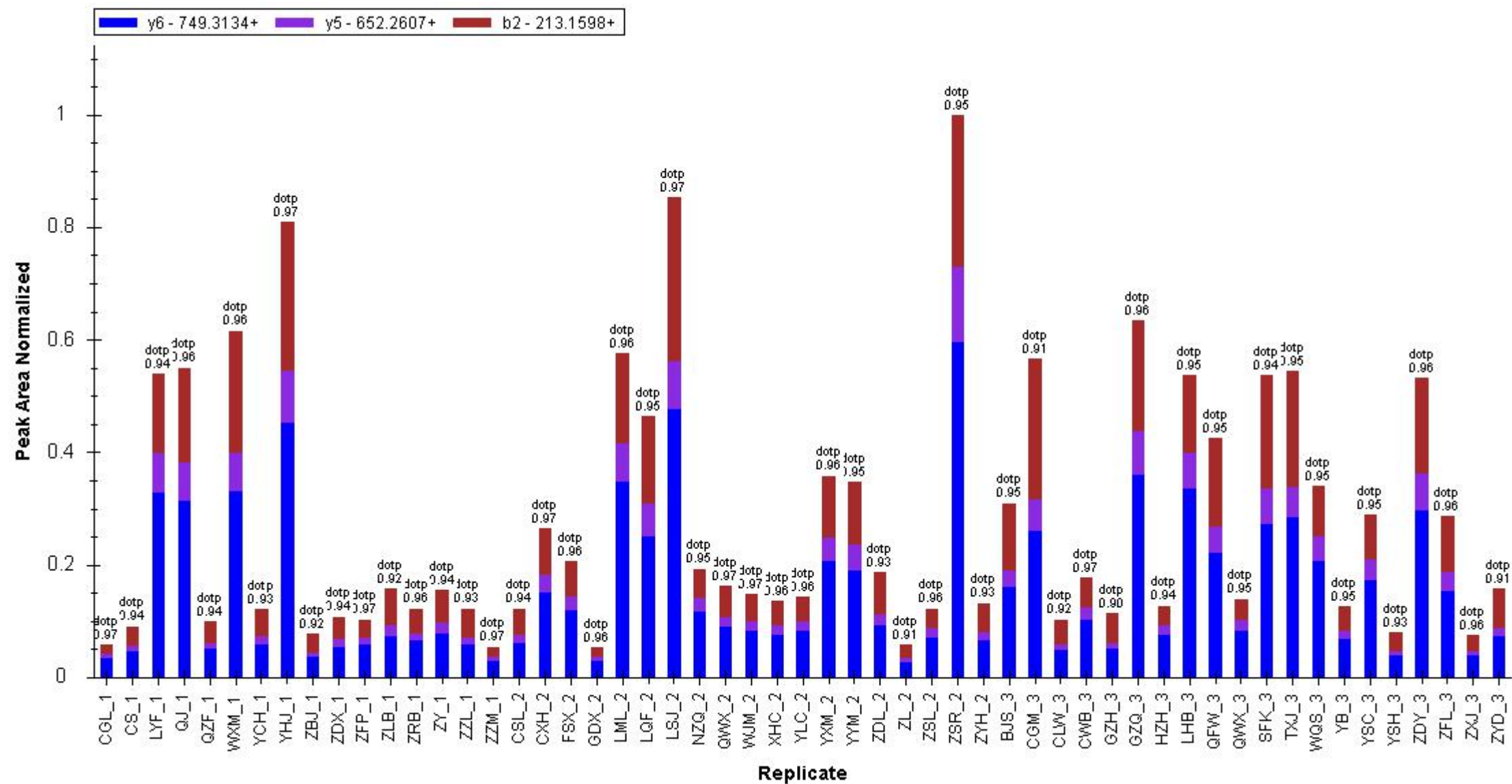

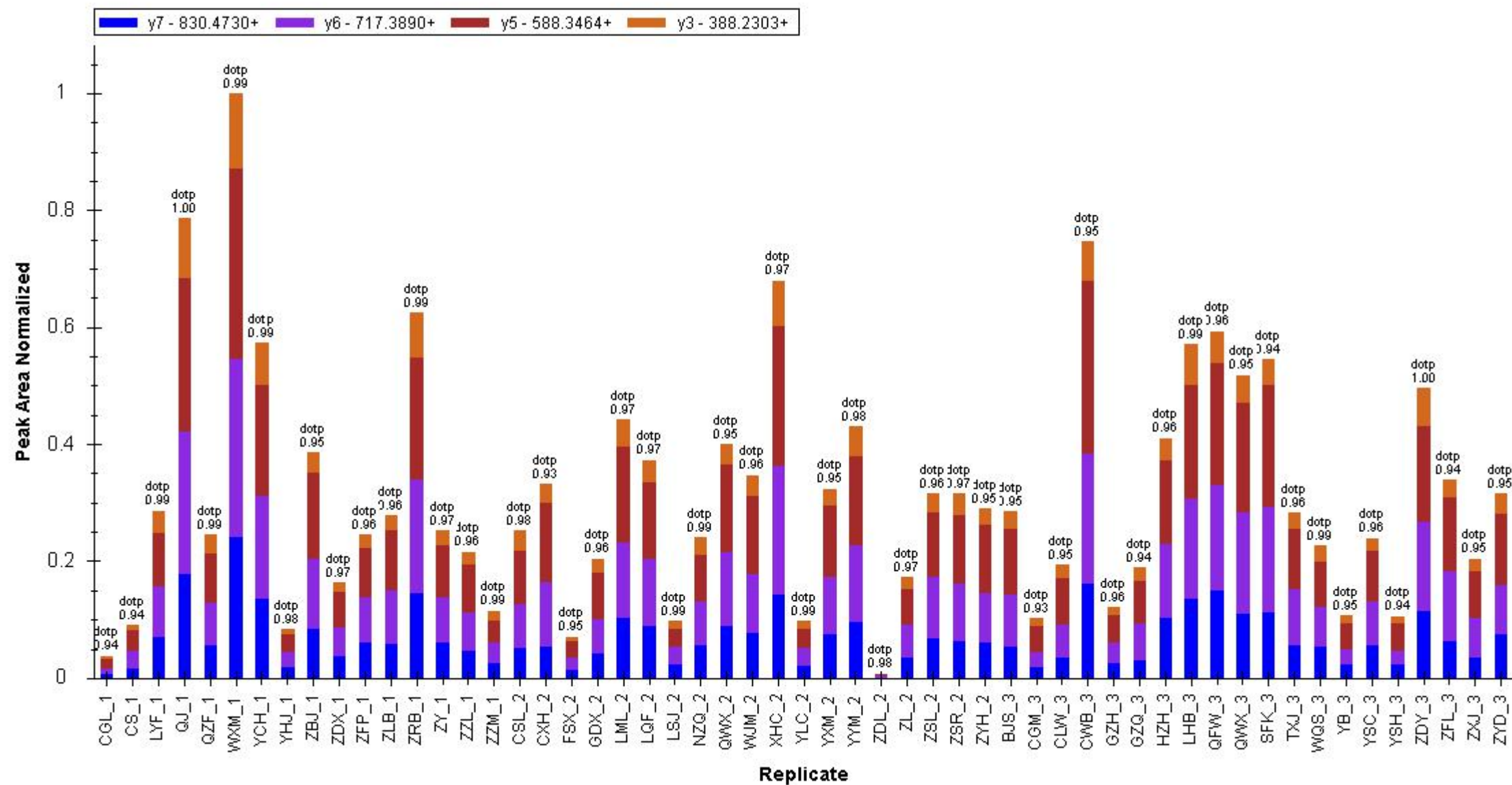

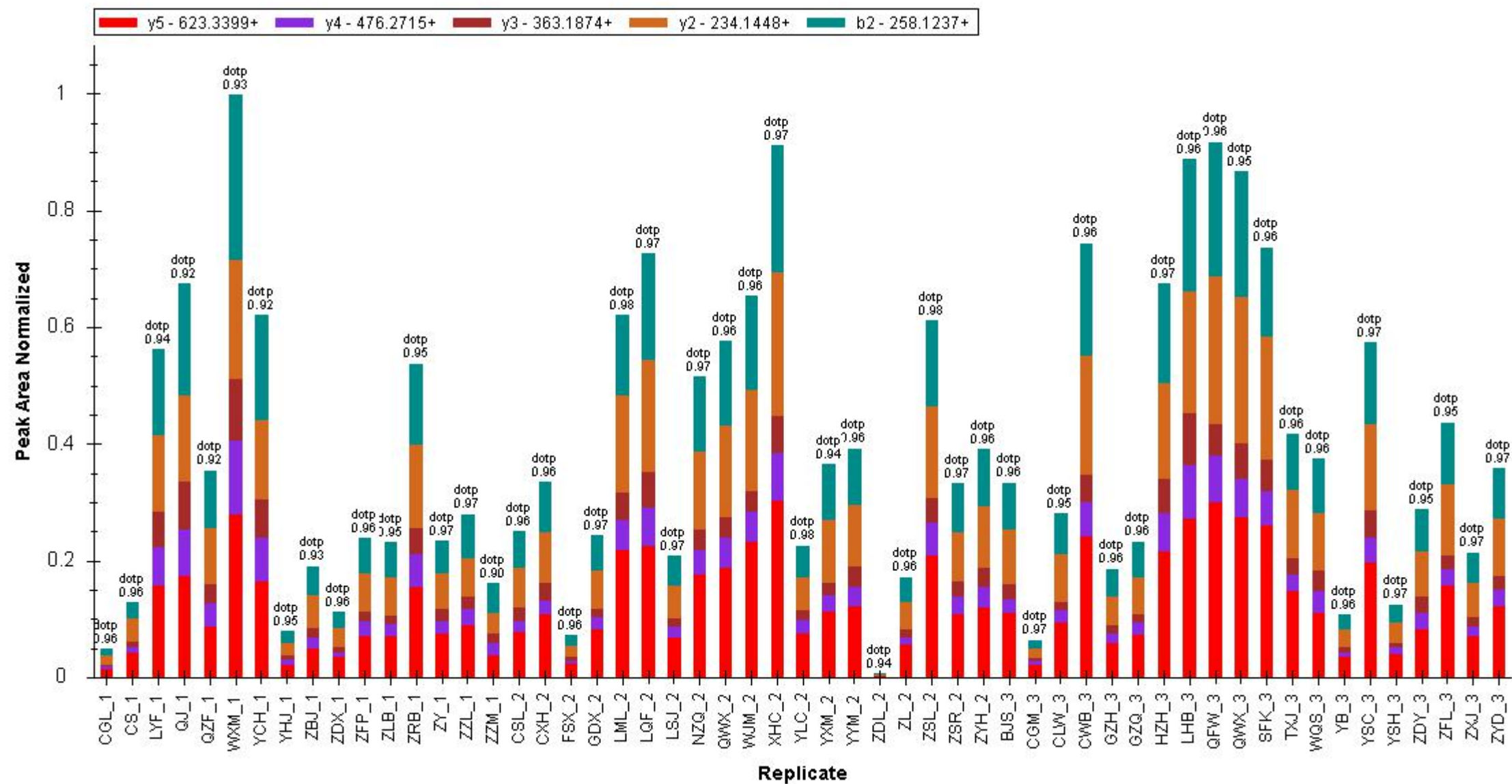

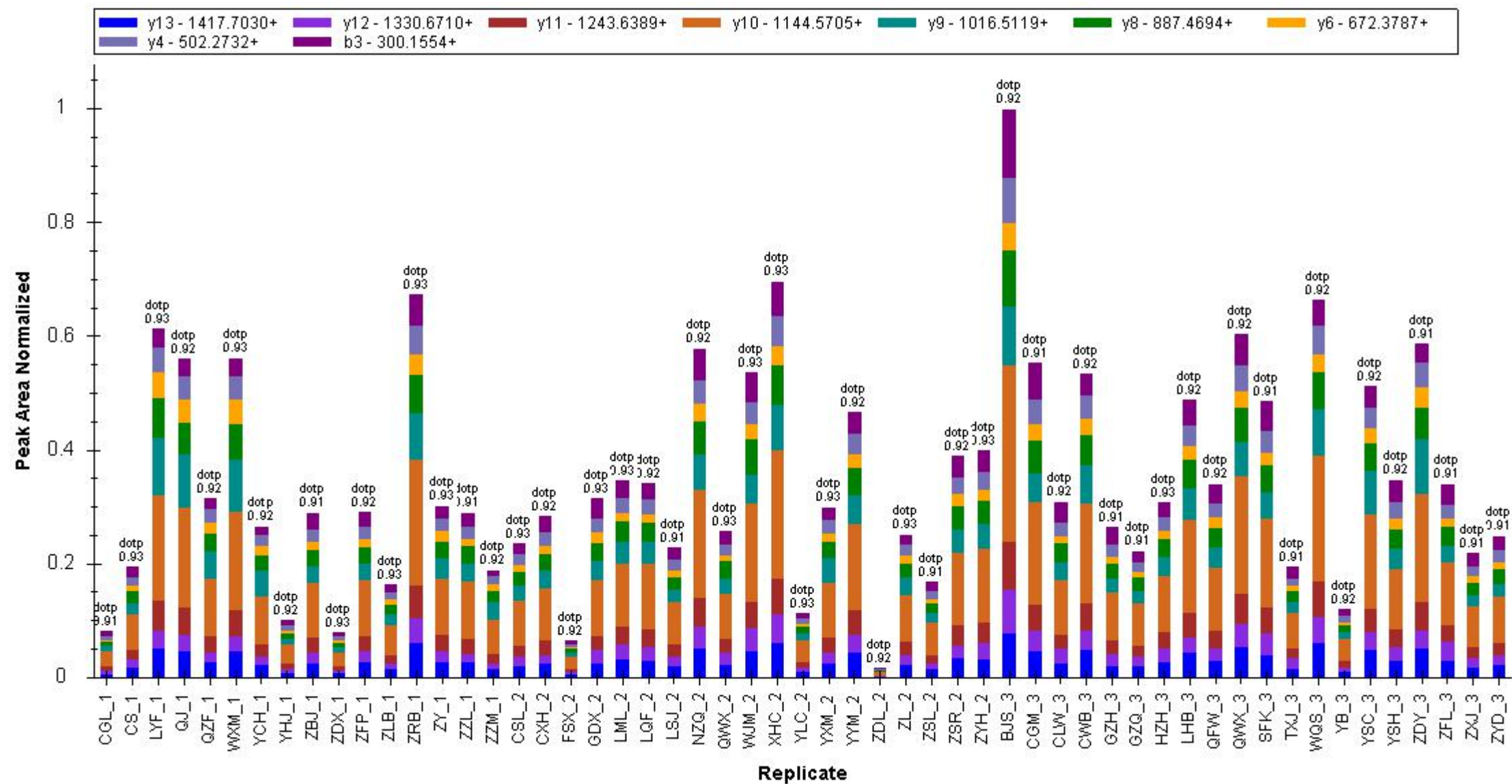

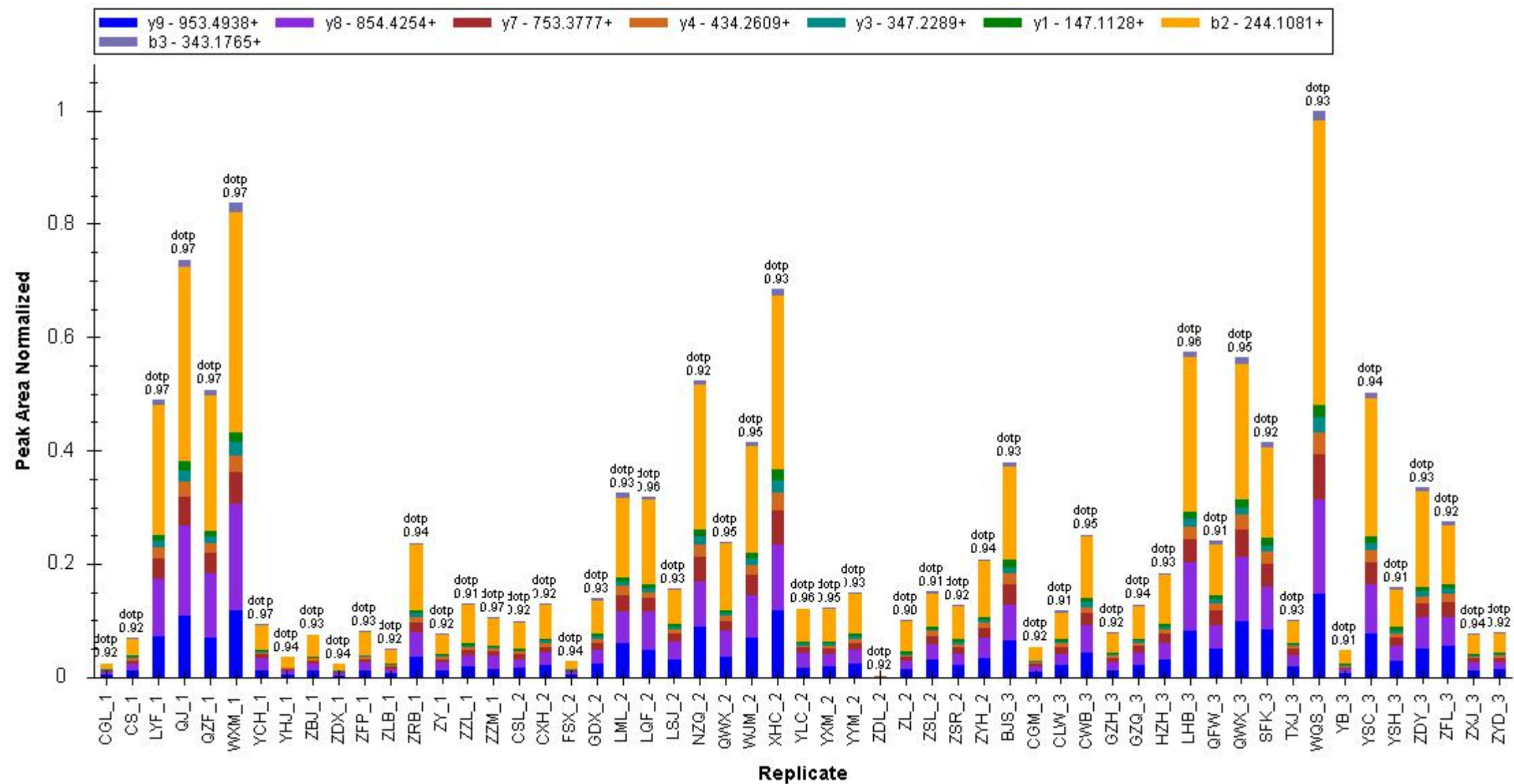

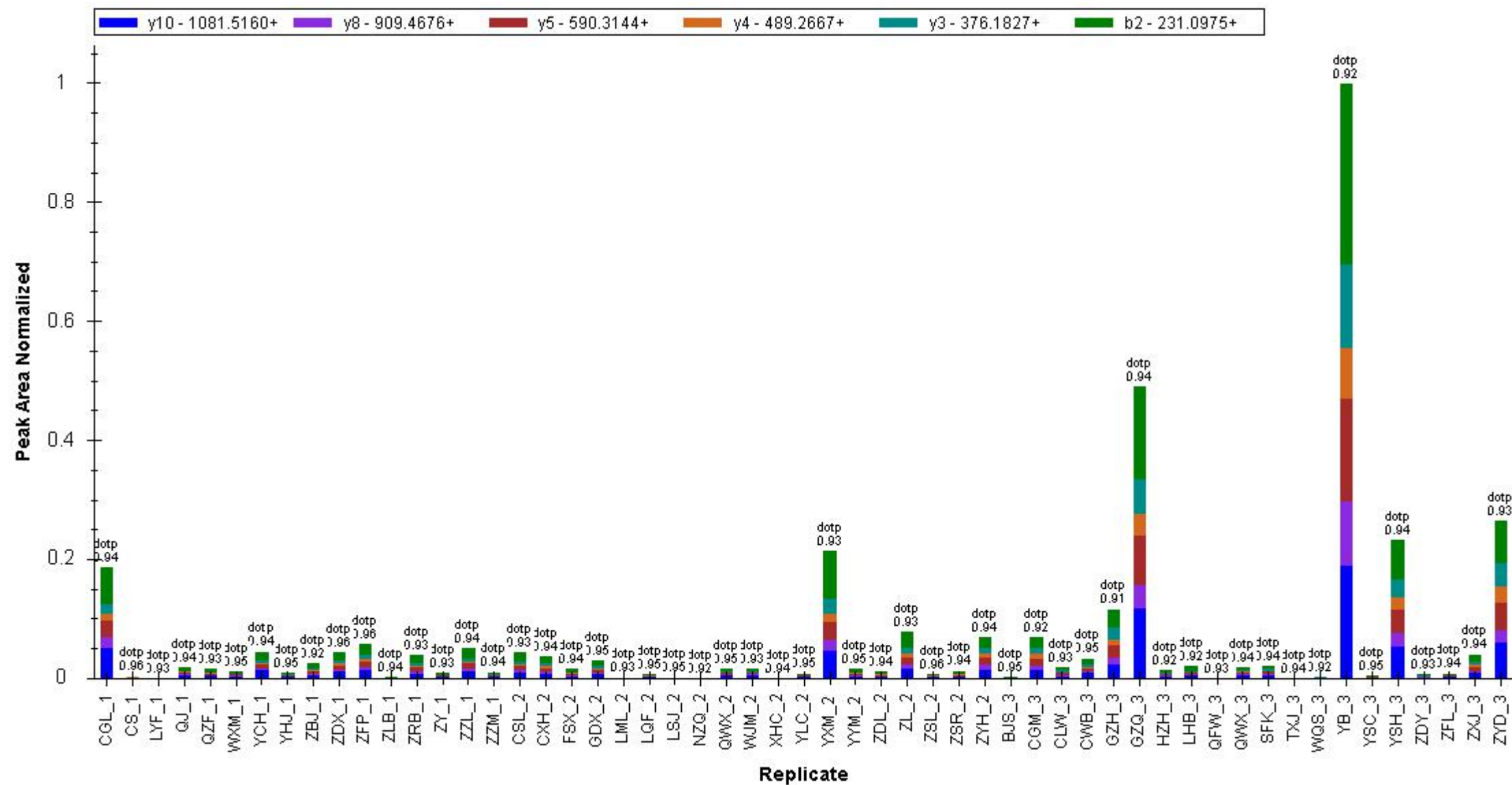

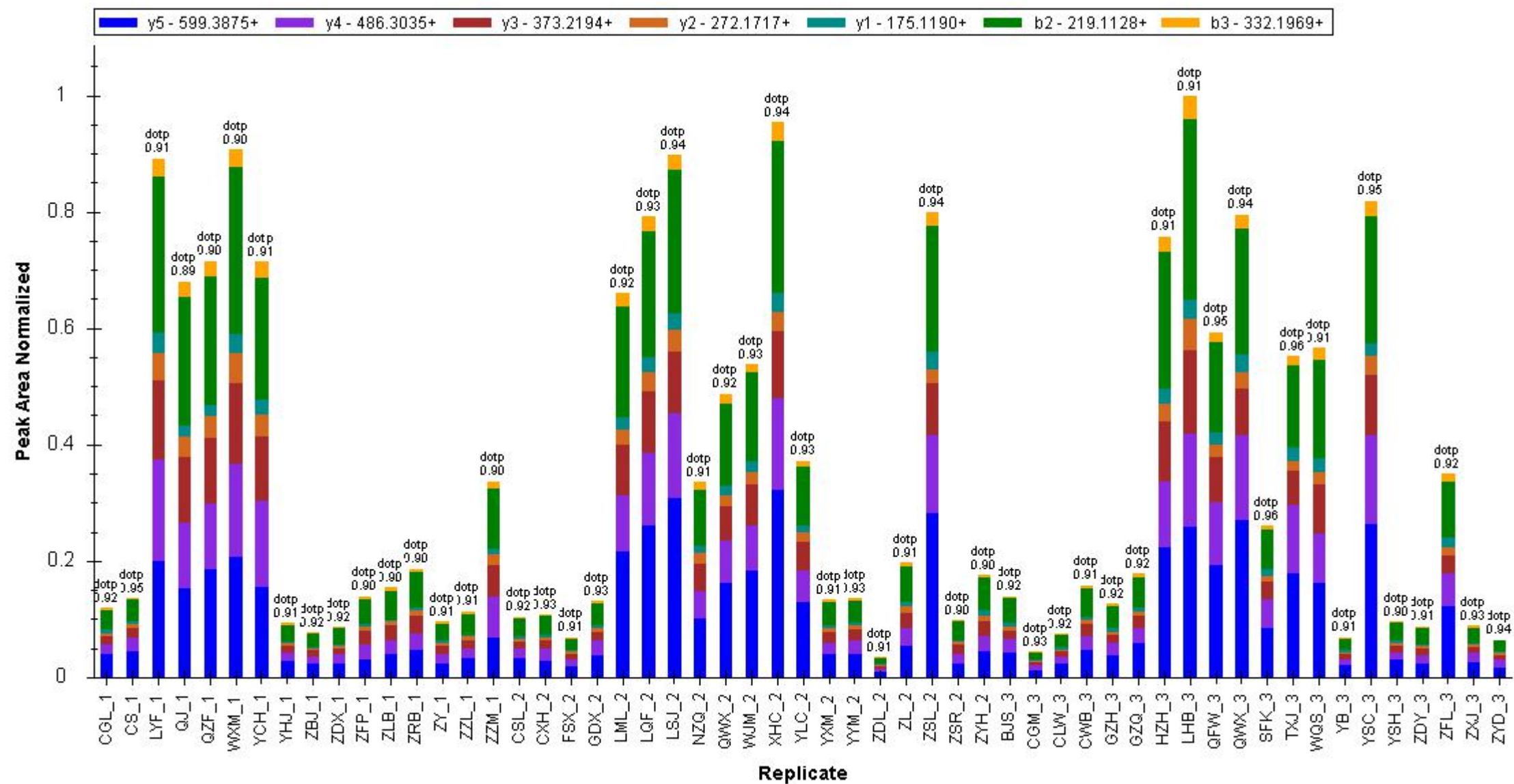

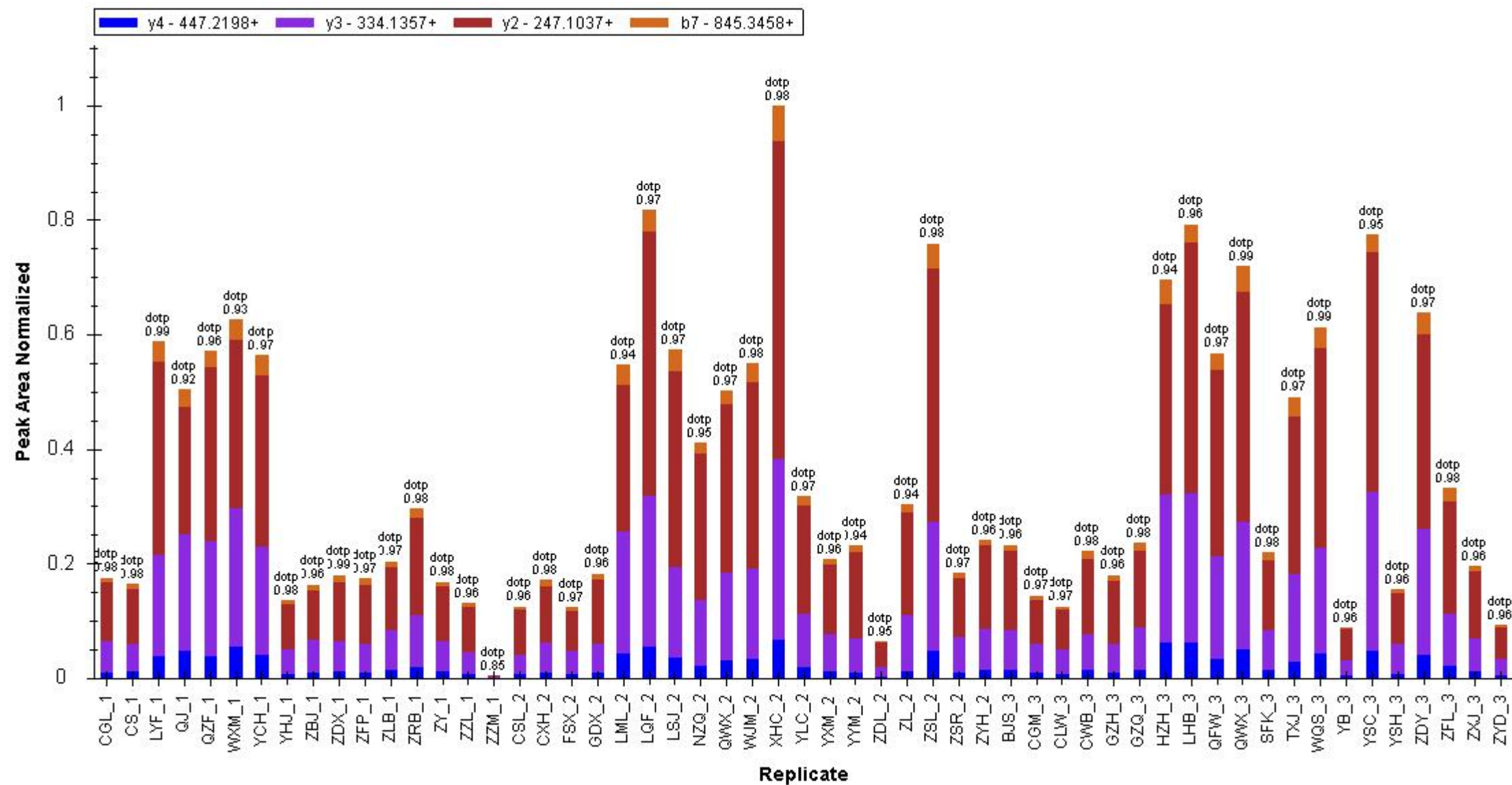

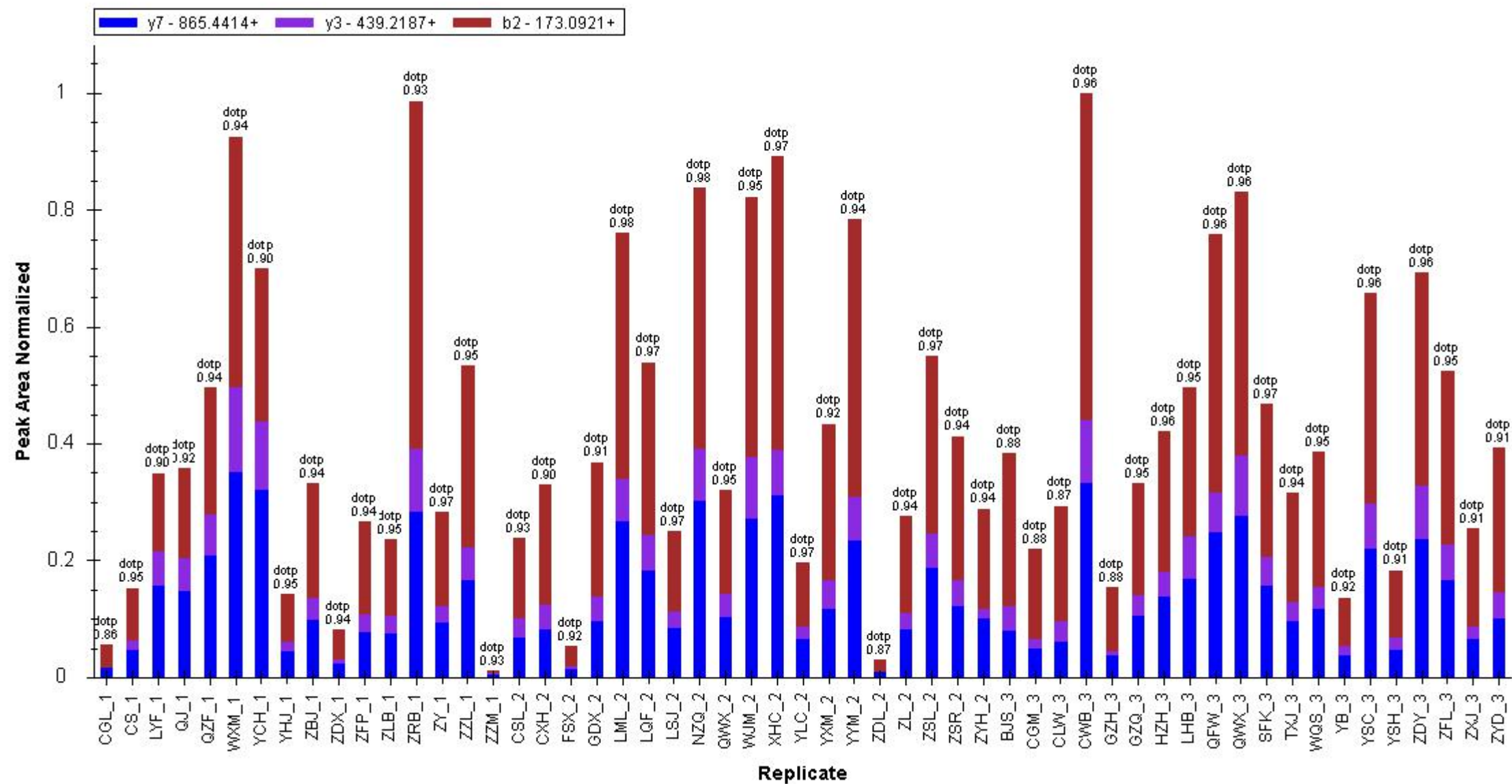

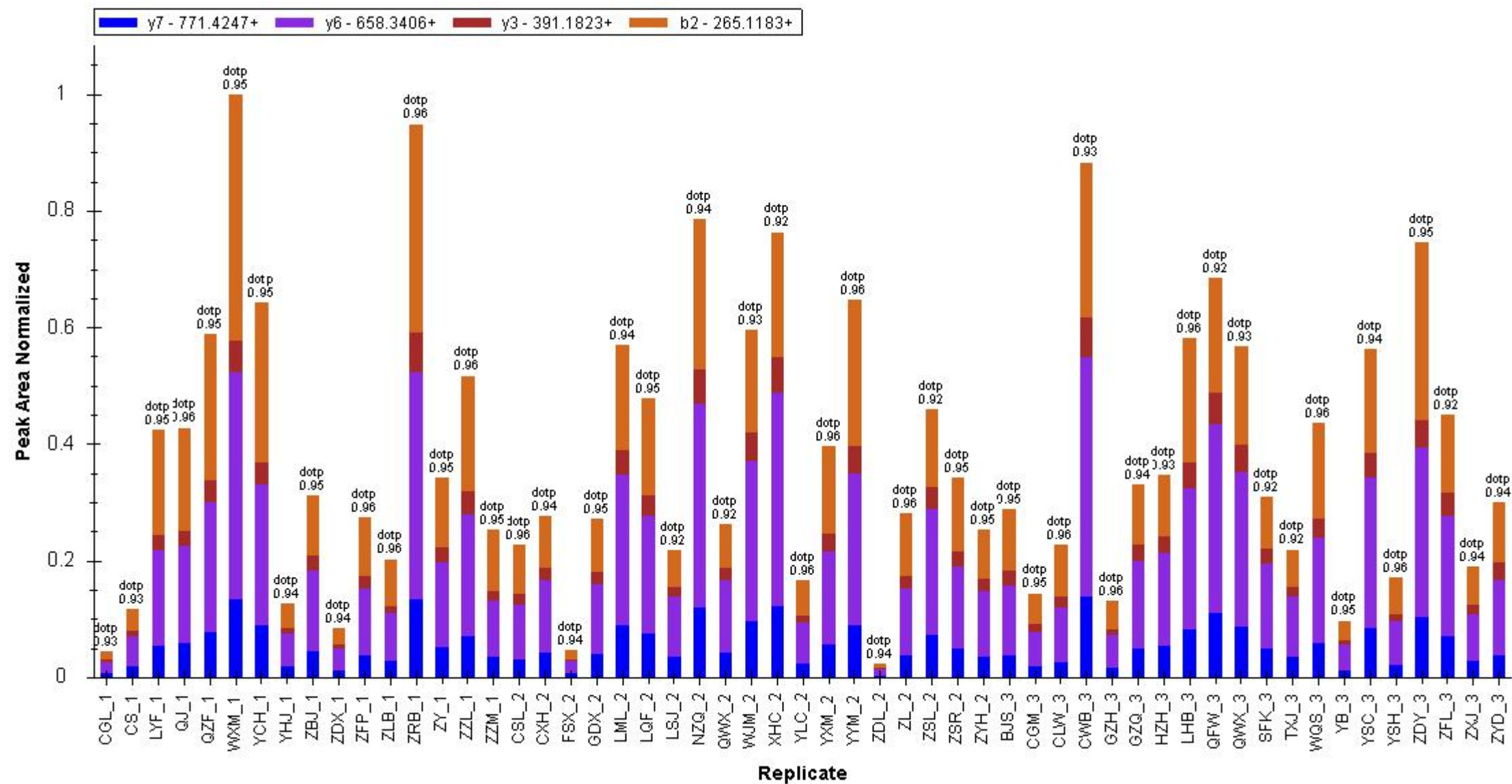

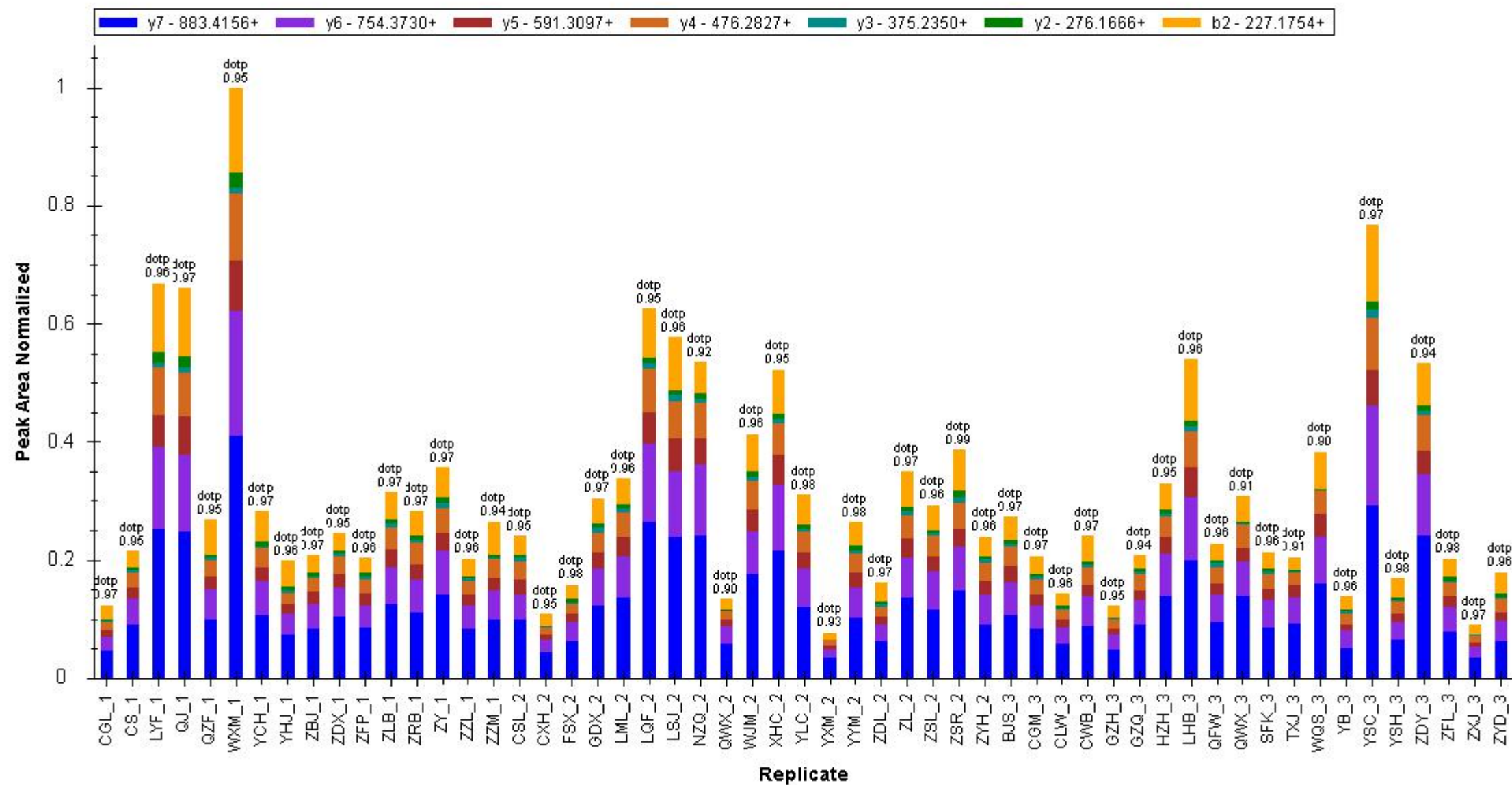

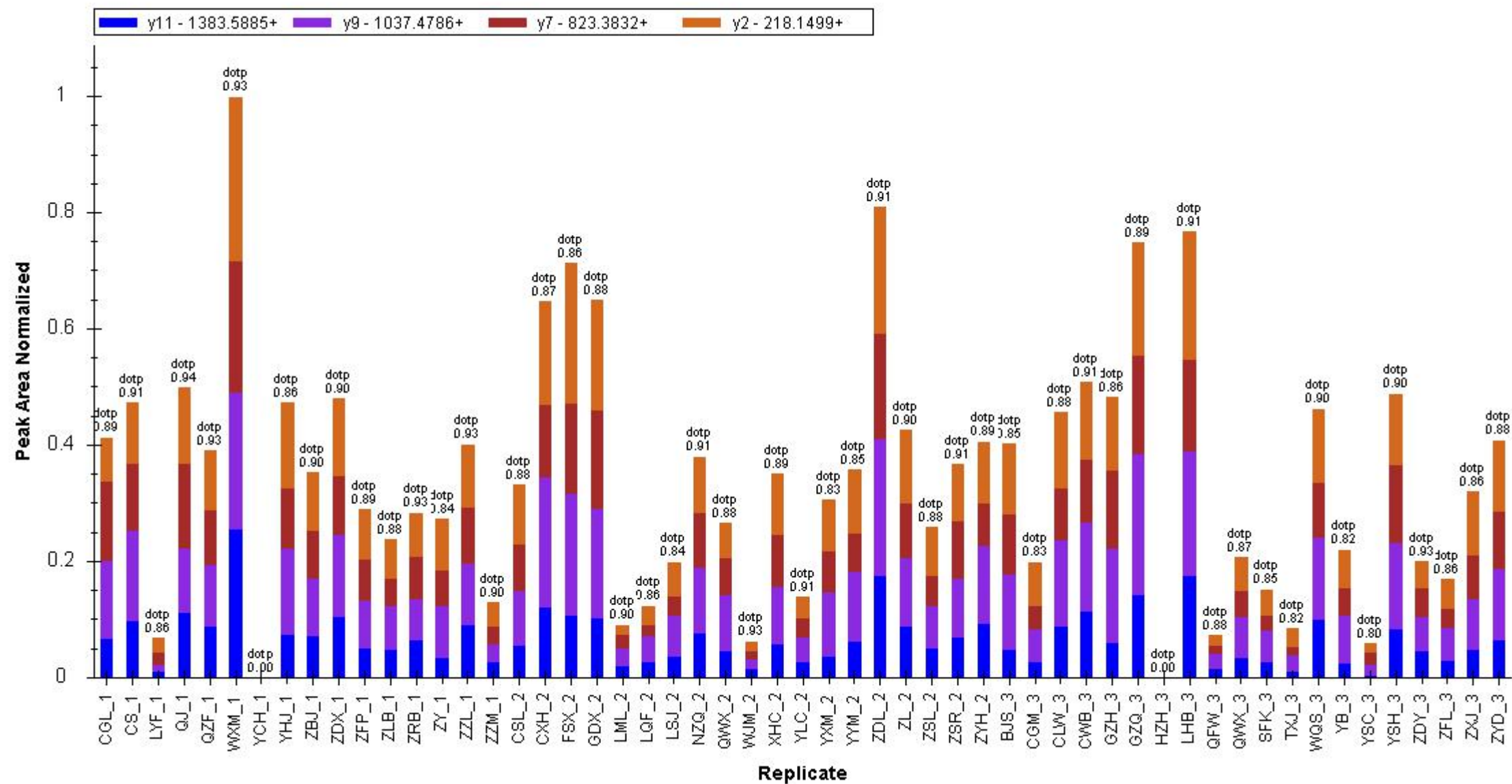

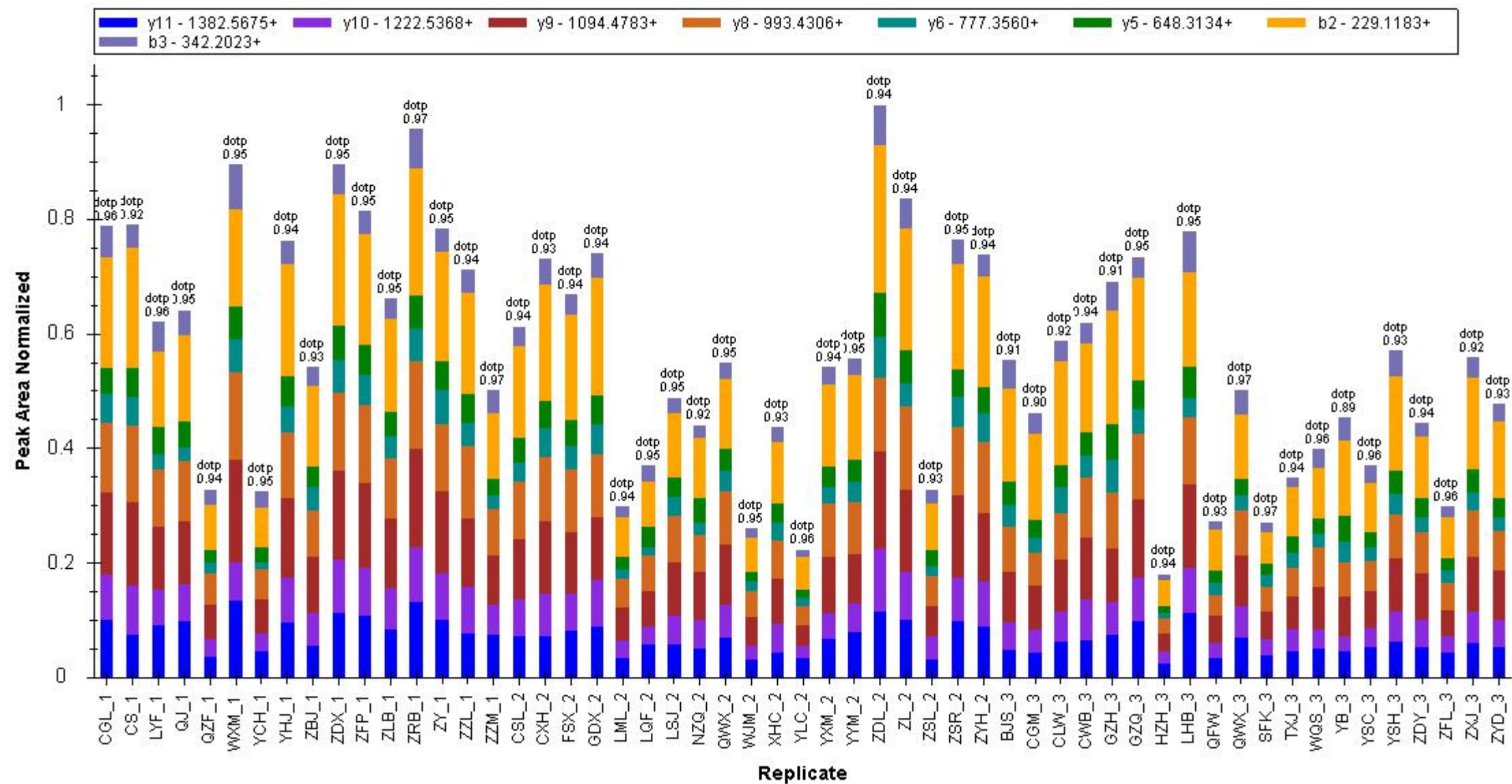

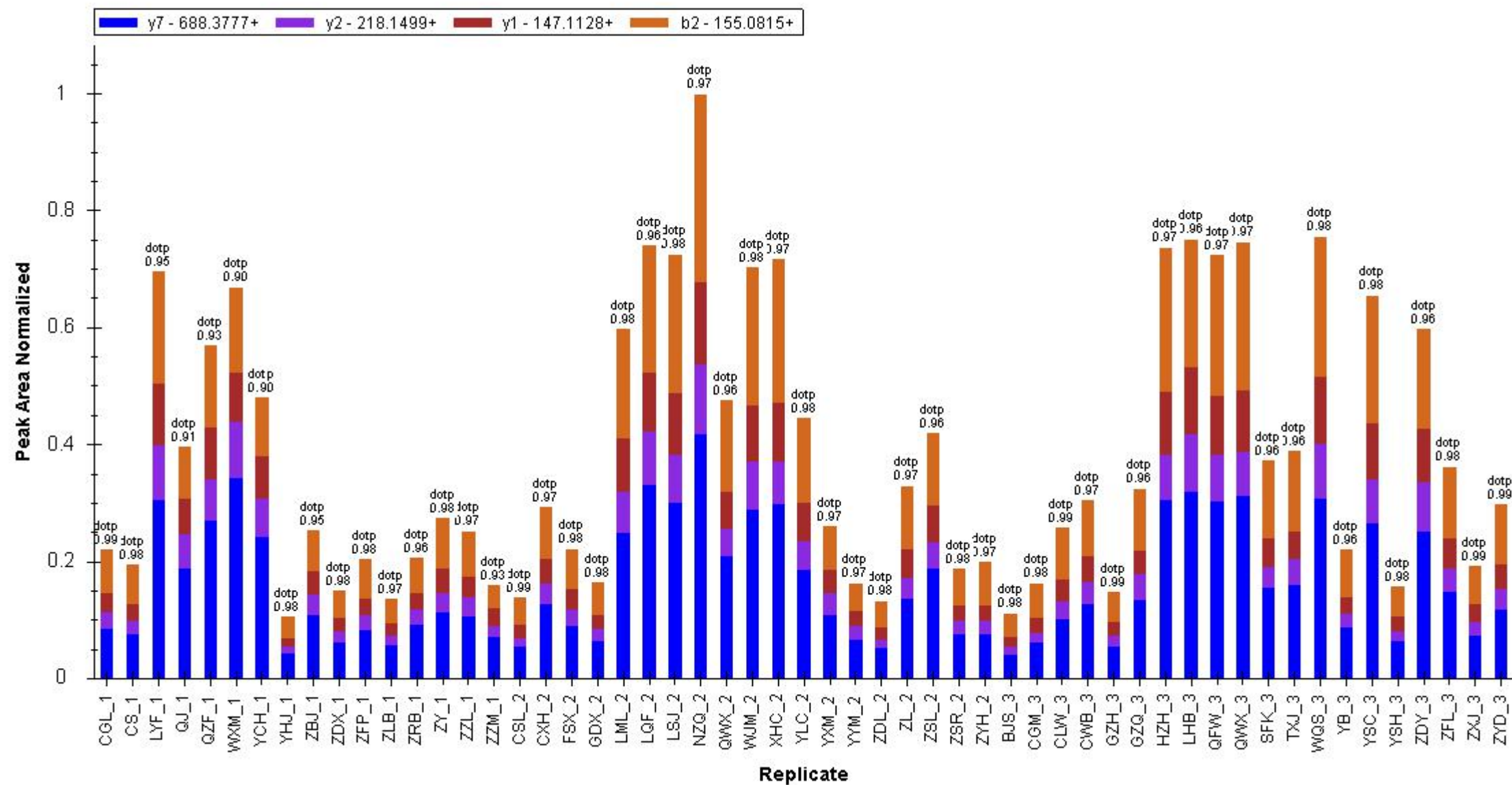

Supplement: Supplementary Figure 2 — The ion peak area distribution of peptide fragment. [file Data_Sheet_2.PDF]
