# Supplementary material for: Investigation of Specific Proteins Related to Different Types of Coronary Atherosclerosis
Source: Front Cardiovasc Med. 2021 Oct 22;8:758035. doi: 10.3389/fcvm.2021.758035 (PMC8569131; doi:10.3389/fcvm.2021.758035)
Supplement: Supplementary Table 1 — Clinical and laboratory characteristics of the experimental group. [file Table_1.DOCX]

**Table1.** Clinical and laboratory characteristics of the experimental group

|  | Healthy people group  N=10 | SCAD  N=10 | t\z\x² | P | SCAD  N=10 | AMI  N=10 | t\z\x² | P | Healthy people group  N=10 | AMI  N=10 | t\z\x² | P |
| --- | --- | --- | --- | --- | --- | --- | --- | --- | --- | --- | --- | --- |
| Na | 143.811±3.183 | 141.784±3.121 | -1.324 | 0.205 | 141.784±3.121 | 142.669±2.381 | 0.676 | 0.509 | 143.811±3.183 | 142.669±2.381 | -0.844 | 0.412 |
| K | 4.159±0.457 | 4.070(3.960-4.460) | -0.096 | 0.923 | 4.070（3.960-4.460） | 4.240（4.100-4.680） | -1.195 | 0.232 | 4.159±0.457 | 4.240（4.100-4.680） | -1.155 | 0.248 |
| Cl | 106.200±2.888 | 105.078±2.236 | -0.902 | 0.381 | 105.078±2.236 | 104.689±1.639 | -0.421 | 0.679 | 106.200±2.888 | 104.689±1.639 | -1.348 | 0.198 |
| Ca | 2.373±0.109 | 2.342±0.120 | -0.541 | 0.596 | 2.342±0.120 | 2.347±0.061 | 0.099 | 0.922 | 2.373±0.109 | 2.347±0.061 | -0.612 | 0.550 |
| P | 1.313±0.197 | 1.119±0.137 | -2.379 | 0.031 | 1.119±0.137 | 1.174±0.251 | 0.583 | 0.568 | 1.313±0.197 | 1.174±0.251 | -1.249 | 0.231 |
| Mg | 0.845（0.808-0.968） | 0.834±0.105 | -1.063 | 0.288 | 0.834±0.105 | 0.844±0.083 | 0.225 | 0.825 | 0.845（0.808-0.968） | 0.844±0.083 | -0.531 | 0.596 |
| urea | 5.259±1.261 | 5.761±1.573 | 0.720 | 0.483 | 5.761±1.573 | 5.260±2.192 | -0.557 | 0.585 | 5.259±1.261 | 5.260±2.192 | 0.001 | 0.999 |
| creatinine | 73.429±9.253 | 75.660(71.835-82.975) | -0.962 | 0.336 | 75.660（71.835-82.975） | 85.067±15.078 | -1.015 | 0.310 | 73.429±9.253 | 85.067±15.078 | 1.886 | 0.079 |
| blood glucose | 5.421±0.815 | 5.886±0.816 | 1.017 | 0.324 | 5.886±0.816 | 7.370±2.280 | 1.573 | 0.134 | 5.421±0.815 | 7.370±2.280 | 2.062 | 0.055 |
| triglyceride | 1.204±0.524 | 1.070(0.965-1.635) | -0.245 | 0.806 | 1.070（0.965-1.635） | 1.960（1.725-2.340） | -1.879 | 0.060 | 1.204±0.524 | 1.960（1.725-2.340） | -2.005 | 0.045 |
| total cholesterol | 4.249±1.120 | 4.464±1.170 | 0.743 | 0.468 | 4.464±1.170 | 3.980（3.685-4.760） | -0.817 | 0.414 | 4.249±1.120 | 3.980（3.685-4.760） | -0.265 | 0.791 |
| high density lipoprotein cholesterol | 1.110±0.292 | 1.126±0.353 | 0.398 | 0.696 | 1.126±0.353 | 0.976±0.184 | -1.090 | 0.291 | 1.110±0.292 | 0.976±0.184 | -0.769 | 0.452 |
| low density lipoprotein cholesterin | 2.818±0.913 | 2.953±0.825 | 0.645 | 0.528 | 2.953±0.825 | 2.630（2.315-3.215） | -0.816 | 0.414 | 2.818±0.913 | 2.630（2.315-3.215） | 0.000 | 1.000 |
| A1 apolipoprotein A1 | 1.085（1.020-1.263） | 1.108±0.300 | -0.287 | 0.774 | 1.108±0.300 | 1.020（0.940-1.235） | -0.204 | 0.838 | 1.085（1.020-1.263） | 1.020（0.940-1.235） | -1.213 | 0.225 |
| B apolipoprotein B | 0.885±0.232 | 0.891±0.227 | 0.389 | 0.702 | 0.891±0.227 | 0.900（0.775-1.050） | -0.245 | 0.806 | 0.885±0.232 | 0.900（0.775-1.050） | -0.567 | 0.571 |
| lipoprotein(a) | 14.154±7.205 | 8.990(3.840-20.940) | -0.490 | 0.624 | 8.990（3.840-20.940） | 6.670（5.040-19.685） | -0.572 | 0.568 | 14.154±7.205 | 6.670（5.040-19.685） | -0.378 | 0.705 |
| Aspartic acid amino converting enzyme | 19.788±3.561 | 29.010(21.975-44.050) | -2.782 | 0.005 | 29.010（21.975-44.050） | 23.341±6.471 | -1.225 | 0.221 | 19.788±3.561 | 23.341±6.471 | 1.373 | 0.188 |
| alanine aminotransferase | 21.044±6.763 | 32.010(22.870-62.050) | -2.163 | 0.031 | 32.010（22.870-62.050） | 30.978±13.462 | -0.735 | 0.462 | 21.044±6.763 | 30.978±13.462 | 2.210 | 0.041 |
| alkaline phosphatase | 87.480±23.947 | 108.396±45.740 | 1.282 | 0.218 | 108.396±45.740 | 93.712±22.697 | -0.952 | 0.354 | 87.480±23.947 | 93.712±22.697 | 0.638 | 0.532 |
| r-glutamyltransferase | 24.933±7.139 | 32.280(19.830-90.800) | -1.280 | 0.200 | 32.280（19.830-90.800） | 49.817±22.818 | -0.327 | 0.744 | 24.933±7.139 | 49.817±22.818 | 2.630 | 0.018 |
| leucine aminopeptidase | 29.341±5.821 | 35.487±8.312 | 1.843 | 0.084 | 35.487±8.312 | 35.444±4.243 | -0.077 | 0.939 | 29.341±5.821 | 35.444±4.243 | 2.690 | 0.016 |
| glutamate dehydrogenase | 6.884±3.585 | 10.302±3.909 | 2.199 | 0.043 | 10.302±3.909 | 10.347±3.044 | -0.188 | 0.853 | 6.884±3.585 | 10.347±3.044 | 2.344 | 0.031 |
| lactic dehydrogenase | 165.595±22.010 | 174.621±33.823 | 0.591 | 0.563 | 174.621±33.823 | 170.020（145.330-220.540） | -0.327 | 0.744 | 165.595±22.010 | 170.020（145.330-220.540） | -0.735 | 0.462 |
| total bilirubin | 14.259±4.098 | 17.050(13.160-21.990) | -1.149 | 0.251 | 17.050（13.160-21.990） | 12.971±4.288 | -1.633 | 0.102 | 14.259±4.098 | 12.971±4.288 | -0.346 | 0.733 |
| direct bilirubin | 2.755±1.016 | 3.170(2.270-4.350) | -1.149 | 0.251 | 3.170（2.270-4.350） | 2.520（1.415-2.935） | -1.470 | 0.141 | 2.755±1.016 | 2.520（1.415-2.935） | -0.286 | 0.775 |
| indirect bilirubin | 11.504±3.154 | 13.880(10.605-18.055) | -1.104 | 0.270 | 13.880（10.605-18.055） | 10.657±3.554 | -1.633 | 0.102 | 11.504±3.154 | 10.657±3.554 | -0.248 | 0.807 |
| cholinesterase | 11893.250±1143.162 | 10360.000(6938.5-11226) | -2.075 | 0.038 | 10360.000（6938.500-11226.000） | 10428.780±1603.589 | -0.572 | 0.568 | 11893.250±1143.162 | 10428.780±1603.589 | -1.724 | 0.103 |
| total bile acid | 4.505(2.185-9.848) | 4.840(4.360-6.245) | -0.221 | 0.825 | 4.840（4.360-6.245） | 3.770±1.929 | -1.551 | 0.121 | 4.505（2.185-9.848） | 3.770±1.929 | -1.470 | 0.142 |
| prealbumin | 282.535±41.152 | 311.310(197.830-325.950) | -0.574· | 0.566 | 311.310（197.830-325.950） | 273.770±48.436 | -0.653 | 0.514 | 282.535±41.152 | 273.770±48.436 | -0.141 | 0.890 |
| total protein | 67.596±6.644 | 66.077±5.879 | -0.154 | 0.880 | 66.077±5.879 | 64.626±2.911 | -0.690 | 0.499 | 67.596±6.644 | 64.626±2.911 | -0.795 | 0.437 |
| Albumin | 42.391±3.183 | 41.290(38.610-43.345) | -0.221 | 0.825 | 41.290（38.610-43.345） | 39.007±2.158 | -1.389 | 0.165 | 42.391±3.183 | 39.007±2.158 | -2.213 | 0.041 |
| globulin | 25.205±4.332 | 25.972±3.669 | 0.641 | 0.531 | 25.972±3.669 | 25.619±2.615 | -0.143 | 0.888 | 25.205±4.332 | 25.619±2.615 | 0.629 | 0.537 |
| ratio of albumin to globulin | 1.719±0.267 | 1.577±0.323 | -1.052 | 0.309 | 1.577±0.323 | 142.669±2.381 | -0.613 | 0.540 | 1.719±0.267 | 1.500（1.390-1.670） | -1.351 | 0.177 |

Note: See Section 2.2 for details of data processing.
