# Supplementary material for: Investigation of Specific Proteins Related to Different Types of Coronary Atherosclerosis
Source: Front Cardiovasc Med. 2021 Oct 22;8:758035. doi: 10.3389/fcvm.2021.758035 (PMC8569131; doi:10.3389/fcvm.2021.758035)
Supplement: Supplementary Table 2 — Clinical and laboratory characteristics of the validation group. [file Table_2.DOCX]

**Table 2.** Clinical and laboratory characteristics of the validation group

|  | SCAD  N=19 | Healthy people group  N=16 | t\z\x² | P | SCAD  N=19 | AMI  N=20 | t\z\x² | P | Healthy people group  N=16 | AMI  N=20 | t\z\x² | P |
| --- | --- | --- | --- | --- | --- | --- | --- | --- | --- | --- | --- | --- |
| Na | 141.767±2.722 | 140.900（140.000-142.160） | -0.488 | 0.625 | 141.767±2.722 | 142.527±2.396 | 0.431 | 0.669 | 140.900(140.000-142.160) | 142.527±2.396 | -1.197 | 0.231 |
| K | 4.248±0.317 | 4.150±0.329 | 0.654 | 0.518 | 4.248±0.317 | 4.210（4.040-4.320） | -0.122 | 0.903 | 4.150±0.329 | 4.210（4.040-4.320） | -0.538 | 0.591 |
| Cl | 105.547±2.194 | 105.993±2.204 | -0.402 | 0.690 | 105.547±2.194 | 104.674±1.876 | -1.503 | 0.142 | 105.993±2.204 | 104.674±1.876 | -1.886 | 0.068 |
| Ca | 2.357±0.062 | 2.329±0.080 | 1.219 | 0.232 | 2.357±0.062 | 2.372±0.074 | 0.563 | 0.577 | 2.329±0.080 | 2.372±0.074 | 1.607 | 0.118 |
| P | 1.191±0.203 | 1.310（1.140-1.380） | -1.393 | 0.163 | 1.191±0.203 | 1.235±0.215 | 0.583 | 0.564 | 1.310（1.140-1.380） | 1.235±0.215 | -0.243 | 0.808 |
| Mg | 0.819±0.076 | 0.875±0.114 | -1.624 | 0.114 | 0.819±0.076 | 0.837±0.078 | 0.627 | 0.535 | 0.875±0.114 | 0.837±0.078 | -1.154 | 0.257 |
| urea | 5.524±1.377 | 4.770（4.320-5.310） | -1.374 | 0.169 | 5.524±1.377 | 5.244±1.783 | -0.740 | 0.464 | 4.770（4.320-5.310） | 5.244±1.783 | -0.434 | 0.665 |
| creatinine | 77.770（72.840-89.105） | 78.960（72.470-87.830） | -0.072 | 0.942 | 77.770(72.840-89.105) | 82.340±12.689 | -0.669 | 0.504 | 78.960（72.470-87.830） | 82.340±12.689 | -0.884 | 0.376 |
| blood glucose | 5.661±0.751 | 5.244±0.467 | 1.981 | 0.057 | 5.662±0.750 | 5.750（4.930-7.830） | -0.526 | 0.599 | 5.244±0.467 | 5.750（4.930-7.830） | -1.733 | 0.083 |
| triglyceride | 1.741±1.071 | 1.738±0.696 | 0.128 | 0.899 | 1.741±1.071 | 1.960（1.700-2.400） | -1.174 | 0.241 | 1.738±0.696 | 1.960（1.700-2.400） | -0.876 | 0.381 |
| total cholesterol | 4.594±1.015 | 4.565±0.925 | 0.327 | 0.746 | 4.594±1.015 | 4.120（3.650-4.760） | -1.036 | 0.300 | 4.565±0.925 | 4.120（3.650-4.760） | -0.366 | 0.716 |
| high density lipoprotein cholesterol | 1.121±0.264 | 1.000（0.950-1.280） | -0.162 | 0.871 | 1.121±0.264 | 0.981±0.221 | -1.704 | 0.097 | 1.000（0.950-1.280） | 0.981±0.221 | -1.179 | 0.238 |
| low density lipoprotein cholesterin | 3.045±0.767 | 3.021±0.808 | 0.305 | 0.763 | 3.045±0.767 | 2.844±1.246 | -0.586 | 0.562 | 3.021±0.808 | 2.844±1.246 | -0.328 | 0.745 |
| A1 apolipoprotein A1 | 1.157±0.213 | 1.164±0.139 | -0.027 | 0.979 | 1.157±0.213 | 1.030（1.000-1.240） | -1.052 | 0.293 | 1.164±0.139 | 1.030（1.000-1.240） | -1.690 | 0.091 |
| B apolipoprotein B | 0.920±0.214 | 0.903±0.230 | 0.457 | 0.651 | 0.920±0.214 | 0.870（0.630-1.030） | -1.189 | 0.234 | 0.903±0.230 | 0.870（0.630-1.030） | -0.366 | 0.714 |
| lipoprotein(a) | 8.990(4.350-20.940) | 11.790(4.160-23.220) | -0.288 | 0.773 | 8.990（4.350-20.940） | 11.080（5.810-15.430） | -0.030 | 0.976 | 11.790（4.160-23.220） | 11.080（5.810-15.430） | -0.239 | 0.811 |
| Aspartic acid amino converting enzyme | 25.070（20.335-30.060） | 20.711±3.893 | -2.663 | 0.008 | 25.070（20.335-30.060） | 23.530（19.690-32.640） | -0.366 | 0.715 | 20.711±3.893 | 23.530（19.690-32.640） | -2.150 | 0.032 |
| alanine aminotransferase | 33.485±16.877 | 26.142±8.403 | 1.524 | 0.138 | 33.485±16.877 | 28.168±11.278 | -1.139 | 0.263 | 26.142±8.403 | 28.168±11.278 | 0.600 | 0.549 |
| alkaline phosphatase | 90.658±27.948 | 80.419±25.707 | 1.073 | 0.292 | 90.658±27.948 | 89.003±18.269 | -0.230 | 0.819 | 80.419±25.707 | 89.003±18.269 | 1.192 | 0.242 |
| r-glutamyltransferase | 38.260(19.830-64.670) | 24.950(20.190-63.670) | -0.397 | 0.692 | 38.260(19.830-64.670) | 47.162±21.825 | -0.427 | 0.670 | 24.950（20.190-63.670） | 47.162±21.825 | -1.367 | 0.172 |
| leucine aminopeptidase | 34.225±6.513 | 33.620(29.230-37.260) | -0.378 | 0.706 | 34.225±6.513 | 33.679±4.625 | -0.303 | 0.764 | 33.620（29.230-37.260） | 33.679±4.625 | -0.033 | 0.973 |
| glutamate dehydrogenase | 10.230(6.870-13.210) | 8.519±4.253 | -1.303 | 0.193 | 10.230(6.870-13.210) | 9.866±3.695 | -0.533 | 0.594 | 8.519±4.253 | 9.866±3.695 | 0.892 | 0.379 |
| lactic dehydrogenase | 170.217±32.913 | 156.840(149.240-187.480) | -0.132 | 0.895 | 170.217±32.913 | 174.560(159.810-189.350) | -1.219 | 0.223 | 156.840(149.240-187.480) | 174.560(159.810-189.350) | -1.483 | 0.138 |
| total bilirubin | 14.559±5.171 | 13.772±4.713 | 0.448 | 0.657 | 14.559±5.171 | 14.118±6.300 | -0.125 | 0.901 | 13.772±4.713 | 14.118±6.300 | 0.287 | 0.776 |
| direct bilirubin | 2.654±0.901 | 2.529±0.819 | 0.410 | 0.685 | 2.654±0.901 | 2.548±1.060 | -0.137 | 0.891 | 2.529±0.819 | 2.548±1.060 | 0.242 | 0.810 |
| indirect bilirubin | 11.905±4.384 | 11.243±3.959 | 0.446 | 0.659 | 11.905±4.384 | 11.571±5.345 | -0.120 | 0.905 | 11.243±3.959 | 11.571±5.345 | 0.290 | 0.774 |
| cholinesterase | 10224.000±1988.071 | 11123.670±1956.842 | -1.287 | 0.208 | 10224.000±1988.071 | 10562.420±1632.560 | 0.342 | 0.735 | 11123.670±1956.842 | 10562.420±1632.560 | -1.121 | 0.271 |
| total bile acid | 4.930（3.755-6.215） | 4.100(2.040-5.210) | -1.378 | 0.168 | 4.930（3.755-6.215） | 3.611±1.849 | -2.057 | 0.040 | 4.100（2.040-5.210） | 3.611±1.849 | -0.600 | 0.548 |
| prealbumin | 306.129±63.511 | 314.360±66.319 | -0.358 | 0.723 | 306.129±63.511 | 284.656±66.997 | -1.013 | 0.318 | 314.360±66.319 | 284.656±66.997 | -1.321 | 0.196 |
| total protein | 66.943±4.274 | 65.650(63.110-71.360) | -0.094 | 0.925 | 66.943±4.274 | 66.026±3.988 | -0.726 | 0.472 | 65.650(63.110-71.360) | 66.026±3.988 | -0.467 | 0.641 |
| Albumin | 41.612±3.461 | 40.890(40.190-43.340) | -0.094 | 0.925 | 41.612±3.461 | 40.528±3.023 | -1.150 | 0.258 | 40.890(40.190-43.340) | 40.528±3.023 | -1.017 | 0.309 |
| globulin | 25.331±3.237 | 25.338±3.412 | -0.006 | 0.995 | 25.331±3.237 | 25.497±2.814 | 0.251 | 0.803 | 25.338±3.412 | 25.497±2.814 | 0.230 | 0.820 |
| ratio of albumin to globulin | 1.670±0.272 | 1.677±0.215 | -0.084 | 0.934 | 1.670±0.272 | 1.612±0.245 | -0.820 | 0.418 | 1.677±0.215 | 1.612±0.245 | -0.971 | 0.339 |

Note: See Section 2.2 for details of data processing.
